# Mechanism of Action of Endophytic Fungi *Hypocrea lixii* and *Beauveria bassiana* in *Phaseolus vulgaris* as Biopesticides against Pea Leafminer and Fall Armyworm

**DOI:** 10.3390/molecules26185694

**Published:** 2021-09-20

**Authors:** Olivia Ngeno Chebet, Leonidah Kerubo Omosa, Sevgan Subramanian, Vaderament-A Nchiozem-Ngnitedem, John Onyari Mmari, Komivi Senyo Akutse

**Affiliations:** 1 Department of Chemistry, University of Nairobi, Nairobi P.O. Box 30197-00100, Kenya; ngenoolivia@gmail.com (O.N.C.); n.vaderamentalexe@gmail.com (V.-A.N.-N.); jonyari@uonbi.ac.ke (J.O.M.); 2 International Centre of Insect Physiology and Ecology (*icipe*), Nairobi P.O. Box 30772-00100, Kenya; ssubramania@icipe.org

**Keywords:** *Hypocrea lixii*, *Beauveria bassiana*, *Liriomyza huidobrensis*, *Spodoptera frugiperda*, defense compounds, volatiles

## Abstract

Endophytic fungal isolates *Hypocrea lixii* F3ST1 and *Beauveria bassiana* G1LU3 were evaluated for their potential to endophytically colonize and induce active compounds in *Phaseolus vulgaris*, as a defense mechanism against pea leafminer (*Liriomyza huidobrensis*) and fall armyworm (*Spodoptera frugiperda*). Endophytic colonization was achieved through seed inoculation with the volatile emissions from *P. vulgaris* plants being analyzed using GC-MS. The crude extracts of *P. vulgaris* obtained using methanol and dichloromethane were assayed against leafminer and fall armyworm larvae using leaf dipping and topical application, respectively. The two isolates successfully colonized the entire host plant (roots, stems, and leaves) with significant variation (*p* < 0.001) between fungal isolates and the controls. The results showed qualitative differences in the volatile profiles between the control plants, endophytically colonized and insect-damaged plants attributed to fungal inoculation and leafminer damage. The crude methanol extracts significantly reduced the percentage pupation of 2nd instar leafminer larvae (*p* < 0.001) and adult-flies emergence (*p* < 0.05). The survival of the 1st instar fall armyworm larvae was also significantly reduced (*p* < 0.001) compared to the controls. This study demonstrated the high potential of endophytic fungi *H. lixii* and *B. bassiana* in inducing mainly specific defense compounds in the common bean *P. vulgaris* that can be used against pea leafminer and fall armyworm.

## 1. Introduction

The common bean, *Phaseolus vulgaris* (Fabaceae), is one of the most important food legumes worldwide [1]. The crops contribute significantly as a valuable source of nutrition and income. However, the production of the common bean is adversely affected by herbivorous pests and the resulting diseases [2]. Among the insect pests of the bean crops, the most destructive is the *Liriomyza* leafminers, a polyphagous invasive species, whose larvae mines under leaf surfaces, creating winding trails on the foliage [3]. Oviposition and mining interfere with nutrient transport and creates avenues for the entry of diseases causing severe yield losses [4]. The quarantine status of *Liriomyza* sp. has also caused export restrictions to international markets and, as a result, loss of revenue [5].

Management and control of leafminers have mainly been through the application of synthetic pesticides. However, there is a growing concern over the continuous use of non-selective chemicals on food crops, the development of resistance, and the potential effects on human health and the environment [6]. To minimize or mitigate and discourage such risks, alternative strategies for pest control that are safe, environmentally friendly, and cost-effective are being encouraged [7,8]. The efficacy of fungal endophytes *Hypocrea lixii* F3ST1 and *Beauveria bassiana* G1LU3 has been demonstrated against the *Liriomyza* leafminers (LMF) using *P. vulgaris* and *Vicia faba* L. (Fabaceae) as host plants [3,9]. Similar systemic endophytic effects were also reported on thrips [10] and bean stem maggots [11]. However, the underlying infection mechanism of the fungus has not been clearly established.

The host plants are usually protected from infection of pests through, reproductive rate reduction [12], feeding deterrence [13], growth retardation [14], and survival and oviposition reduction [15]. Induced plant resistance to arthropod herbivores could be attributed to the production of defense chemical compounds [16]. Fungal endophytes have exoenzymes necessary for colonization of the hosts and the consequent metabolic interactions [17]. Endophytic fungi have been previously reported to stimulate natural defense mechanisms including biosynthesis and accumulation of phytoalexins [18,19]. Metabolic interactions of fungal endophytes and the host plant encourage the synthesis of bioactive secondary compounds such as alkaloids that are toxic to pests at larval stages [20]. β-Phellandrene is a cyclic monoterpene reported to have an attractive ability for the general insect predator *Macrolophus pygmaeus* [21]. Camphor is a terpene identified in volatile oils from *Cinnanomum camphora* and Ocimum plants. It has been shown to have insect repellence [22] and insect attractant abilities [23]. Plant volatiles containing terpinen-4-ol, a naturally occurring monoterpene, have been reported to affect feeding and oviposition of *Thrips tabaci* Lindeman (Thysanoptera: Thripidae) [24]. Induction of defense compounds has also been previously associated with abiotic factors such as wounding. Leafminer larval wounding has been reported to induce the production of green leaf volatiles (esters, aldehydes, and alcohols), nitrogen-containing compounds, and terpenoids [25]. Volatile compounds have also been emitted by plants wounded by leaf-eating spider mites, sucking insects and caterpillars including (*Z*)-3-hexenal, (*E*)-2-hexenal, (*Z*)-3- hexen-l-ol, (*Z*)-3-hexen-1-yl acetate, linalool, (3*E*)-4,8-dimethyl-1,3,7-nonatriene, indole, α-trans-bergamotene, β-farnesene, (*E*)-nerolidol, (3*E*,7*E*)-4,8,12-trimethyl-1,3,7,11-6 tridecatetraene. Production of these volatile defense compounds by plants involves three biosynthetic pathways: the shikimic acid pathway for methyl salicylate, the isoprenoid pathway for terpenoids, and the fatty acid/lipoxygenase pathway for green leaf volatiles [26]. Menjivar et al., have reported metabolic accumulation in the tomato due to endophytic inoculation of the fungus *Fusarium oxysporum* [18]. Wei and co-workers also detected terpenes and oximes in volatiles emitted by bean plants as a response to the attack by agromyzid flies [25]. This study, therefore, investigated the induction of defense compounds by endophytic fungi to reduce leafminer attack on the common bean and to determine the potency of obtained extracts against *Liriomyza huidobrensis* (Diptera: Agromyzidae) and fall armyworm (FAW), *Spodoptera frugiperda* (Lepidoptera: Noctuidae).

## 2. Results

### 2.1. Colonization Assessment of Phaseolus vulgaris Plants Inoculated with Hypocrea lixii F3ST1 and Beauveria bassiana G1LU3

**Figure 1:**
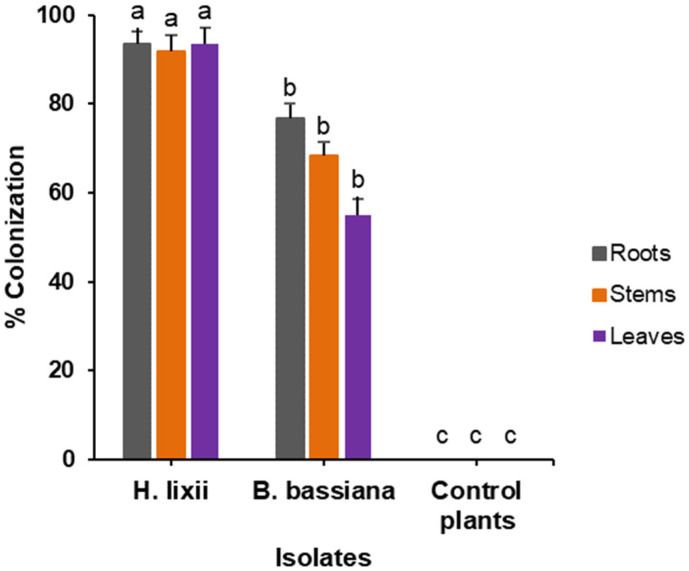
Percentage colonization of leaves, stems and roots of *Phaseolus vulgaris* plants by endophytic isolates of *Hypocrea lixii* F3ST1 and *Beauveria bassiana* G1LU3. Means with the same letter are not significantly different using a generalized linear model (GLM) (*p* < 0.001).

Viability tests for the harvested conidia from the fungal cultures exhibited germination of more than 90% for both isolates. The two isolates, *H. lixii* F3ST1 and *B. bassiana* G1LU3 successfully colonized the host plant *P. vulgaris*. The endophytic colonization of the common bean host plant in the leaves, stems, and roots varied significantly (*p* < 0.001) between fungal isolates and the controls (Figure 1). *Hypocrea lixii* F3ST1 exhibited the highest percentage of colonization in the leaves (93.3%) and stems (91.6%), while *B. bassiana* G1LU3 had the highest percentage of colonization in the roots (76.6%), moderate in the stems (68.3%), and lowest in the leaves (55.0%). No colonization was observed in the control plants.

### 2.2. Organic Metabolites Characterized from Phaseolus vulgaris Plants

**Figure 2:**
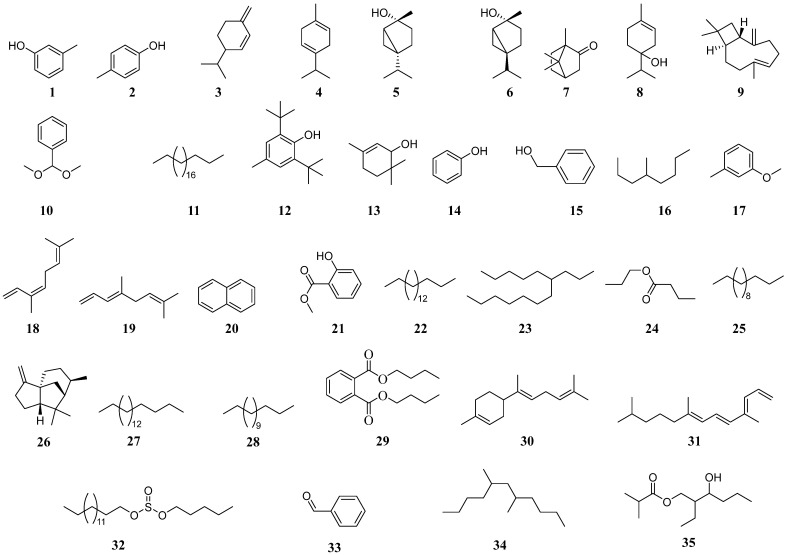
GC-MS analysis of volatiles characterized from *Phaseolus vulgaris*.

The emitted volatiles detected from control *P. vulgaris*, leafminer damaged, fungal inoculated, and inoculated damaged plants, showed considerable variations in the number of compounds produced. The GC-MS chromatograms indicated two volatiles (*m*-cresol (**1**) and *p*-cresol (**2**)) in control *P. vulgaris* emissions, ten (β-phellandrene (**3**), α-terpinene (**4**), *cis*-sabinene hydrate (**5**), *trans*-sabinene hydrate (**6**), camphor (**7**), terpinen-4-ol (**8**), (*E*)-caryophyllene (**9**) benzaldehyde dimethyl acetal (**10**), heneicosane (**11**), and butylated hydroxytoluene (**12**)) in leafminer damaged plants, Table 1 and Figure 2. There were also qualitative variations in volatile emissions between control bean plants and fungal-inoculated plants. The GC-MS profiles showed five (*m*-cresol (**1**), *p*-cresol (**2**), *cis*-1,1,3,5-tetramethyl cyclohexane (**13**), phenol (**14**), and benzyl alcohol (**15**)) in *H. lixii* inoculated plants and nineteen (*p*-cresol (**2**), (*E*)-caryophyllene (**9**), benzaldehyde dimethyl acetal (**10**), heneicosane (**11**) butylated hydroxytoluene (**12**), 4-methyl octane (**16**), 3-methylanisole (**17**), (*Z*)-β-ocimene (**18**), (*E*)-β-ocimene (**19**), naphthalene (**20**), methyl salicylate (**21**), heptadecane (**22**), 6-propyl tridecane (**23**), propyl butanoate (**24**), tridecane (**25**), α-cedrene (**26**), octadecane (**27**), tetradecane (**28**), and dibutyl phthalate (**29**)) in *H. lixii* inoculated leafminer-damaged plants. *Beauveria bassiana* inoculated plants emitted the highest number of compounds. Sixteen volatiles (*p*-cresol (**2**), (*E*)-caryophyllene (**9**), benzaldehyde dimethyl acetal (**10**), heneicosane (**11**), butylated hydroxytoluene (**12**), 3-methylanisole (**17**), (*Z*)-β-ocimene (**18**), (*E*)-β-ocimene (**19**), naphthalene (**20**), methyl salicylate (**21**), α-cedrene (**26**), tetradecane (**28**), dibutyl phthalate (**29**), (*E*)-γ-bisabolene (**30**), 4,8,12-trimethyl-1,3*E*,7*E*,11-tridecatetraene (**31**), and sulfurous acid pentyl undecyl ester (**32**)) in *B. bassiana* inoculated bean plants. The GC-MS chromatogram revealed more than thirty (30) peak signals of which, eleven volatiles (α-terpinene (**4**), *cis*-sabinene hydrate (**5**), camphor (**7**), terpinene-4-ol (**8**), benzaldehyde dimethyl acetal (**10**), heneicosane (**11**) butylated hydroxytoluene (**12**), α-cedrene (**26**), benzaldehyde (**33**), 5,7-dimethyl undecane (**34**), and 2-methyl-2-ethyl-3-hydroxyhexylpropanoic acid (**35**)) were consistently released by leafminer-damaged *B. bassiana* inoculated plants, Table 1 and Figure 2 (Figures S1–S35, Supporting Information).

### 2.3. Effects of Plant Extracts on Pupation of 2nd Instar Liriomyza huidobrensis Larvae

**Figure 3:**
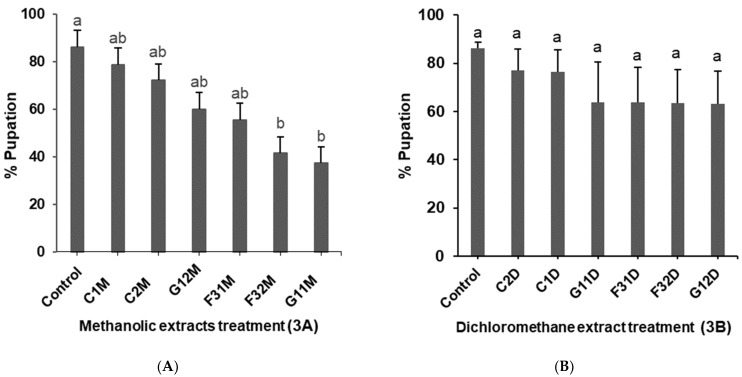
(**A**): Effect of *Phaseolus vulgaris* plant methanol extracts on the pupation of 2nd instar *Liriomyza huidobrensis* larvae. (**B**): Effect of *Phaseolus vulgaris* plant dichloromethane extracts on the pupation of 2nd instar *Liriomyza huidobrensis* larvae. Bars denote means ± standard error at 95% CI means with the same letter are not significantly different. (Control): Tween 80 solution, (C1M): Control plants methanol extract, (C2M): Control plants exposed to insects methanol extract, (G12M): *B. bassiana* inoculated plants exposed to insects methanol extracts, (F31M): *H. lixii* inoculated plants methanol extract, (F32M): *H. lixii* inoculated plants exposed to insects methanol extracts, (G11M): *B. bassiana* inoculated plants methanol extract, (C2D): Control plants exposed to insects dichloromethane extract, (C1D): Control plants dichloromethane extract, (G11D): *B. bassiana* inoculated plants dichloromethane extract, (F31D): *H. lixii* inoculated plants dichloromethane extract, (F32D): *H. lixii* inoculated plants exposed to insects dichloromethane extracts, (G12D): *B. bassiana* inoculated plants exposed to insects dichloromethane extracts.

Fewer leafminer pupae emerged from the 2nd instar leafminer larvae dipped in endophytically colonized plant extracts compared to the controls (Figure 3A). There were significant differences in the pupation from the larvae dipped into the methanol extracts (*p* < 0.001) (Figure 3A). The percentage pupation of larvae dipped in *B. bassiana* inoculated plant methanol extract (G11M) and *H. lixii* inoculated plants exposed to insects’ methanol extract (F32M) was lowest with percentage pupation of 37 and 41%, respectively, compared to 86% in the controls (Figure 3A). However, dichloromethane extracts did not show significant differences in the pupation of the 2nd instar leaf miner (*p* = 0.2) among the treatments (Figure 3B).

### 2.4. Effects of Plant Extracts on Emergence of Liriomyza huidobrensis Adult Flies

**Figure 4:**
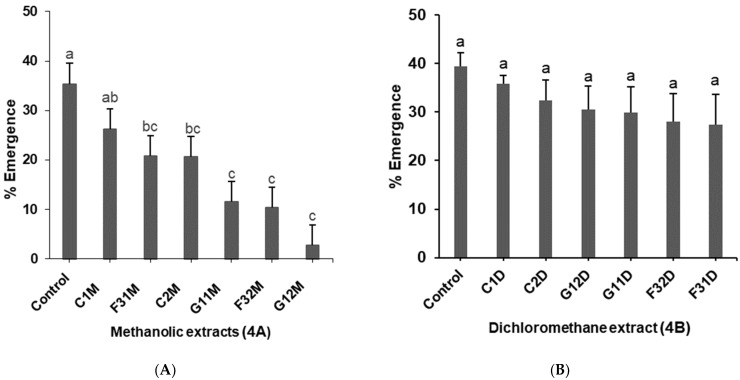
(**A**). Effect of *Phaseolus vulgaris* plant methanolic extracts on adult emergence of 2nd instar *Liriomyza huidobrensis* larvae. (**B**). Effect of *Phaseolus vulgaris* plant dichloromethane extracts on adult emergence of 2nd instar *Liriomyza huidobrensis* larvae. Bars denote means ± standard error at 95% CI means with the same letter are not significantly different. (Control): Tween 80 solution, (C1M): Control plants methanol extract, (F31M): *H. lixii* inoculated plants methanol extract, (C2M): Control plants exposed to insects methanol extract, (G11M): *B. bassiana* inoculated plants methanol extract, (F32M): *H. lixii* inoculated plants exposed to insects methanol extracts, (G12M): *B. bassiana* inoculated plants exposed to insects methanol ex-tracts, (C1D): Control plants dichloromethane extract, (C2D): Control plants exposed to insects dichloromethane extract, (G12D): *B. bassiana* inoculated plants exposed to insects dichloromethane extracts, (G11D): *B. bassiana* inoculated plants dichloromethane extract, (F32D): *H. lixii* inoculated plants exposed to insects dichloromethane extracts, (F31D): *H. lixii* inoculated plants dichloromethane extract.

**Figure 5:**
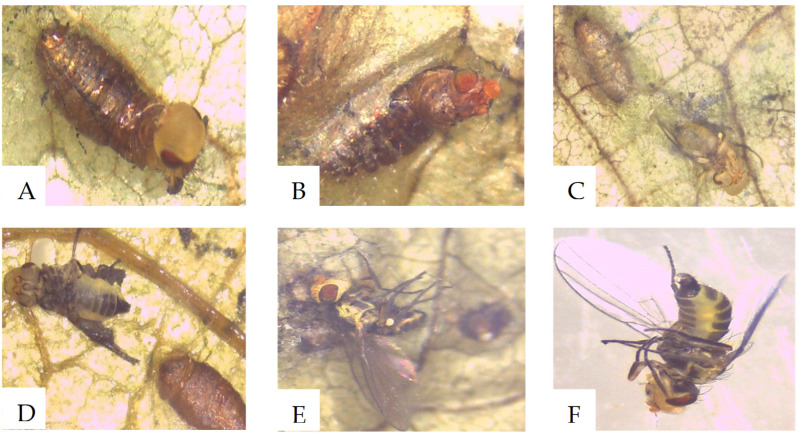
Effects of fungi colonized *Phaseolus vulgaris* plant extracts on *Liriomyza huidobrensis* pupae, (**A**)—insects were stuck (whole part of the body) inside their pupal cases, (**B**)—insects died inside the pupae, (**C**)—Insects failed to emerge, (**D**–**F**)—Insects died before they could fully emerge.

There were significant differences in the adult emergence from larvae dipped into methanol extracts (*p* < 0.05) compared to the controls. From the methanol extract, only a few flies emerged from G11M, F32M, and G12M treatments (Figure 4A). The number of adult flies that emerged from larvae dipped in *B. bassiana* inoculated plant methanol extracts (G11M), *H. lixii* inoculated plants exposed to insects’ methanol extracts (F32M) and *B. bassiana* inoculated plants exposed to insects methanol extracts (G12M) had the lowest percentages of emergence (2, 10 and 11%, respectively) (Figure 4A). However, dichloromethane extracts did not show any significant differences in the emergence of the 2nd instar leaf miner (*p* = 0.2) (Figure 4B). Most flies in the controls emerged successfully while flies treated in extracts failed to emerge because most of them were stuck in the pupal cases (Figure 5).

### 2.5. Effects of Fungal and Plant Extracts on 1st Instar Fall armyworm, Spodoptera frugiperda Larvae

**Figure 6:**
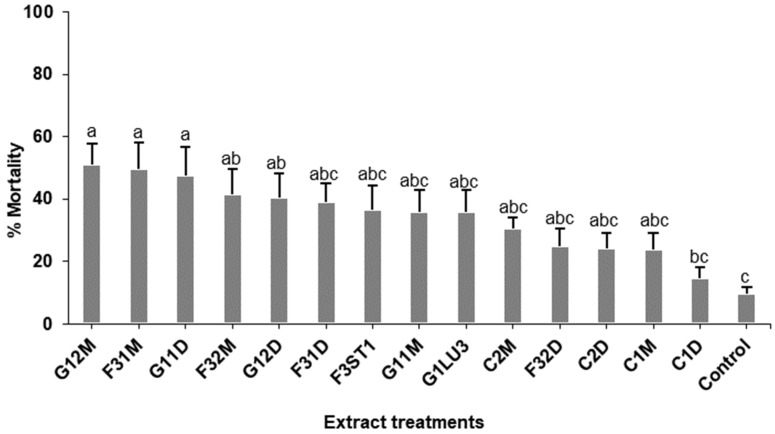
Effect of *Phaseolus vulgaris* plant extracts on the mortality of 1st instar fall armyworm, *Spodoptera frugiperda* larvae. Bars denote means ± standard error at 95% CI (*p* = 0.05). Means with the same letter are not significantly different. (G12M): *B. bassiana* inoculated plants exposed to insects methanol extracts, (F31M): *H. lixii* inoculated plants methanol extract, (G11D): *B. bassiana* inoculated plants dichloromethane extract, (F32M): *H. lixii* inoculated plants exposed to insects methanol extracts, (G12D): *B. bassiana* inoculated plants exposed to insects dichloromethane extracts, (F31D): *H. lixii* inoculated plants dichloromethane extract, (F3ST1): *H. lixii* fungal extract, (G11M): *B. bassiana* inoculated plants methanol extract, (G1LU3): *B. bassiana* fungal extract, (C2M): Control plants exposed to insects methanol extract, (F32D): *H. lixii* inoculated plants exposed to insects dichloromethane extracts, (C2D): Control plants exposed to insects dichloromethane extract, (C1M): Control plants methanol extract, (C1D): Control plants dichloromethane extract, (Control): Tween 80 solution.

The survival of 1st instar fall armyworm larvae dipped into inoculated plant extracts was significantly reduced (*p* < 0.0001) as compared to the controls (Figure 6). Mortality of up to 51% was observed in the treatments within 7 days compared to 9.5% in the controls. The mortality of 1st instar fall armyworm (FAW) larvae dipped in inoculated plant extracts varied among treatments (*p* < 0.0001). At 7 days post-treatment, mean mortality was 50.9% for *B. bassiana* inoculated leafminer damaged plant methanol extract (G12M), 49.5% for *H. lixii* inoculated plant methanol extracts (F31M) and 47.6% for *B. bassiana* inoculated plant dichloromethane extract (G11D) compared to 9.5% in the controls. There were significant differences in the mortality of 1st instar fall armyworm dipped in methanol extracts (*p* = 0.001) and dichloromethane extracts (*p* = 0.008) compared to controls. There were significant differences in the mortality rates of 1st instar FAW dipped into non-exposed plant extracts (*p* = 0.001) and exposed plant extracts (*p* = 0.002). Methanolic extracts of *B. bassiana* inoculated plants was the most lethal with 4.42 days median lethal time (LT_50_), 4.50 days in *H. lixii* inoculated plant methanol extracts (F31M) and 4.72 days in *B. bassiana* inoculated plant dichloromethane extract (G11D) compared to >10 days in the controls (Table 2).

## 3. Discussion

The two isolates, *H. lixii* F3ST1 and *B. bassiana* G1LU3 successfully colonized the entire *P. vulgaris* host plants (roots, stems, and leaves) through seed inoculation. However, *H. lixii* F3STI exhibited the highest percentage of colonization of the various plant parts as compared to *B. bassiana* G1LU3. Similar findings were reported by Akutse et al. [9] with *H. lixii* and *B. bassiana*. Akello and Sikora [12] also showed that *B. bassiana* (one of the isolates used in the current research), colonized maize plants. Moreover, this finding was similar to the results of Akutse et al. [9] where *Vicia faba* as host plant indicated substantial variations in the colonization of the different parts of the plants [9]. The colonization of the various plant pieces reveals that the fungus inoculum migrates inside the plant system from the inoculated point to the other parts of the host plant. This can be observed in *B. bassiana* G1LU3 where the endophyte colonized less the leaves as compared to the roots and stems. The differences in the level of colonization of different parts of the plants among the fungal isolates are consistent with results reported by Akello and Sikora [12], and Gathage et al. [3]. The cause of higher levels of colonization in the stems and leaves is not apparent but could indicate disparities in physiological and microbial environments in the distinct plant parts. Petrini and Fisher [27] reported that endophytic fungi showed tissue specificity because they are modified to certain special environments present in the allotted plant parts.

The emitted volatiles detected from control *P. vulgaris* plants, insect (LMF)-damaged plants, fungi-inoculated plants, and inoculated damaged plants, showed considerable variations in the number of compounds produced. Only two volatiles were identified in control emissions *m*-cresol (**1**) and *p*-cresol (**2**). These compounds were identified in both controls and endophytically colonized plants. This implies that cresol compounds are produced by the common bean plants in both endophytes colonized and non-colonized plants. Cresol compounds have previously been proven to elicit induced systemic resistance against disease pathogens [28].

The difference in volatile emissions by endophyte-free bean plants when damaged by the insect indicates a response due to puncturing and wounding or development of mines by leafminer larvae. This is related to cascade defense responses triggered by leafminer larval feeding. A puncture caused by leafminer larvae results in a large mine development that extends towards the base of the leaf. Plant wounding has previously been shown to result in reactions such as the hydrolysis of cyanogenic glycoside to produce insect-toxic prussic acid [29]. Wei et al. [25] have also reported the production of defense volatiles when the common bean plants are damaged by both pests and artificial methods (mechanical injuries).

Production of compounds by *H. lixii* inoculated plants that were not present in control plants suggest production of volatiles as a result of *H. lixii* fungal colonization for defense. One of the compounds identified from *H. lixii* inoculated plants was a polymethylated cycloalkane known as *cis*-1,1,3,5-tetramethyl cyclohexane (**13**). It has been reported among anti-fungal volatiles produced by non-pathogenic fungi *Fusarium oxysporum* [30]. The compound phenol (**14**) produced by dicotyledonous plants are phytoalexins that disrupt the cell structure and metabolism of fungal pathogens [19]. The compound benzyl alcohol (**15**) which was identified from *H. lixii* inoculated plants is an aromatic alcohol previously isolated from tomatoes, fruits, and tea. These aroma components in fruits are responsible for attracting pollinators and seed dispersers and also strengthening plant defense responses [31].

Qualitative differences are also evidenced among *H. lixii* inoculated plants and *H. lixii* inoculated leafminer-damaged plants. A combination of both inoculated and insect damage allowed for more changes in plant metabolism. Compounds previously linked to insect damage were also produced ((*E*)-caryophyllene (**9**), benzaldehyde dimethyl acetal (**10**), heneicosane (**11**), and butylated hydroxytoluene (**12**). More changes were also evident in the production of compounds not previously linked to either induction (4-methyl octane (**16**), 3-methylanisole (**17**), (*Z*)-β-ocimene (**18**), (*E*)-β-ocimene (**19**), naphthalene (**20**), methyl salicylate (**21**), heptadecane (**22**), 6-propyl tridecane (**23**), propyl butanoate (**24**), tridecane (**25**), α-cedrene (**26**), octadecane (**27**), tetradecane (**28**) and dibutyl phthalate (**29**). Production of compounds by *H. lixii* inoculated and damaged leafminer plants that were not present in control plants suggest induction as a result of both insect damage and fungal colonization. The compound 4-methyloctane (**16**) emitted by *H. lixii* inoculated and leafminer-damaged plants has been previously identified in headspace volatile samples of various plants including the grape fruit with reports of involvement in resistance to fungus *Botrytis cinerea* [32]. The compound, 3-methylanisole (**17**) released by both *H. lixii* and *B. bassiana* inoculated plants is a methoxy toluene, a microbial volatile that has been previously extracted from both fungi and bacteria. It has been shown to reduce pine weevil attraction to the pine host plant [33]. The compounds (*Z*)-β-ocimene (**18**) and (*E*)-β-ocimene (**19**) in inoculated plants are monoterpenes found in various plants and fruits. Spider mites have been shown to induce emission of (*E*)-β-Ocimene (**19**) from the *Lotus japonicus* for defense [34]. The compound naphthalene (**20**) was also in inoculated and insect-damaged plants and is the simplest polycyclic aromatic hydrocarbon. Naphthalene (**20**) is an insect repellent that has been shown to be produced by fungal endophytes [35]. The compound methyl salicylate (**21**) has been shown to be an insect-induced volatile [36]. It has also been found in endophyte colonized plants [37]. The compound α-cedrene (**26**) is a sesquiterpene metabolite that has been identified in several plant metabolites including cedar and citrus [38]. Volatile constituents from endophytic fungi constituting cedrene have been reported to have anti-fungal and anti-bacterial activities [39].

Although *H. lixii* inoculated plants consistently released volatiles absent in control plant emissions, their quantities were considerably lower compared to volatiles emitted by plants inoculated with *B. bassiana. Beauveria bassiana* inoculated plants produced the highest number of detected emissions. A plant’s response to one fungus is therefore not similar to another fungus. Synthesis of biologically active compounds through induction by the microorganisms is attributed to metabolic interactions between the fungal endophytes and their host plant [40]. Fungal endophytes have exoenzymes necessary for colonization of the hosts and their metabolic interactions that include an increase in the plant’s defense metabolites to balance their association [17]. Induced systemic resistance in endophytically colonized plants is due to the activation or production of mycotoxins. *Hypocrea lixii*, which exhibited higher colonization percentages, produced lower levels of volatiles compared to *B. bassiana* inoculated plants. This suggests that the interaction between the two fungi and the *P. vulgaris* host plant differed, and the consequent metabolism also differed.

Changes in metabolism due to both insect damage and inoculation were also evident with *B. bassiana* inoculated plants. The compound benzaldehyde (**33**) was reported in volatile organic compounds in *Vitis vinifera* as an outcome of induced metabolic changes in the host plant by the arbuscular mycorrhizal fungus *Funneliformis mosseae* [41]. The alkane, dimethyl undecane (**34**) has been previously found in fungal volatile constituents of the *Monilinia* species [42]. The compound 2-methyl-2-ethyl-3-hydroxyhexyl (**35**) propanoate is among the flavor compounds produced by yeast [43]. An insect repellent, produced by all plant treatments except control plants and *H. lixii* inoculated plants, benzaldehyde dimethyl acetal (**10**) was the most abundant at 16% for insect-damaged *B. bassiana* inoculated plants. Another major constituent of the blend being terpinen-4-ol (**8**) at 16%. It has previously been identified in blends with insect predator attractive abilities. Natural enemies including insect predators, attack a broad variety of herbivorous insects and are drawn by these chemical cues. Walling, [16] has shown that a specialist parasitoid could differentiate its host caterpillar from a non-host caterpillar based on specific volatile blends of host plants induced by caterpillars ((*Z*)-3-hexenal, (*E*)-2-hexenal, (*Z*)-3- hexen-l-ol, (*Z*)-3-hexen-1-yl acetate, linalool, (3*E*)-4,8-dimethyl-1,3,7-nonatriene, indole, a-trans-bergamotene, (*E*)-,β-farnesene, (*E*)-nerolidol, (3*E*,7*E*)-4,8,12-trimethyl-1,3,7,11-6 tridecatetraene). The compounds identified in the study include volatiles previously identified as predator attractants. Volatiles induced by leafminer-damaged bean plants and *H. lixii* and *B. bassiana* fungi inoculated plants, therefore, have the potential to attract parasitoids as a defense against herbivore attack. Two species of parasitic wasp, *Diglyphus isaea* and *Opius dissitus* parasitize *Liriomyza* leafminer larvae [20]. Zhao et al. [20] noted that adults of *Diglyphus isaea* orientate in the direction of plant smells correlated with *Liriomyza sativae*-infected plants. Specific compounds from complex herbivore-induced volatile blends are proven to play a part in the discriminatory foraging behaviour of their natural enemies [44]. Menjivar et al. [18] also reported variation in quality and quantity of solvent extracts between control tomato plants and tomato plants with *Trichoderma atroviride* strain MT-20, *T. atroviride* strain S-2, and *Fusarium oxysporum* strain 162 implying changes in metabolism as a result of fungal colonization. Menjivar et al. [18] reported metabolic accumulation in tomato leaves due to endophytic inoculation. Colonization of the host plant *P. vulgaris*, therefore, triggers the production of compounds for defense against herbivorous insects including the leafminer. The compounds deter feeding and oviposition of the pest through insect repellence and predator attraction. The toxic compounds also infect the pest through effects on its physiology. However, the identities of compounds were based on comparisons of mass spectra available from an MS library. Therefore, some of the identifications may be tentative especially for volatiles with *trans-cis* isomers.

Adult flies failed to emerge from their pupal skins in the pupae that were affected by extract treatment. This points to the potent compounds produced by the plant with insecticidal activity that were not previously produced by non-colonized plants. Similar results were reported on LMF exposed to plants inoculated with *H. lixii* F3ST1 and *B. bassiana* G1LU3 [8]. Induction of defense compounds and accumulation of metabolites in the plants allows for deterrence of leafminer feeding and effects on the development of its larvae as the mechanism behind previously reported infection. The profiled bioactive compounds could be potentially used as pest control agents against the insect. In addition to pupal mortality, plant extracts inoculated with fungal endophytes reduced the survival of the FAW larvae. This suggests that the key compounds produced by the endophytically colonized plants are also potentially lethal to other insect pests and their larvae for further protection of the plant. The identified compounds could therefore be further tested on their effects against various pests for their sustainable management. Some endophytes have the ability to produce the same or similar bioactive compounds as those that originated from their host plants. Zhao et al. [45] investigated the progress of endophytic fungi to produce plant-derived bioactive compounds such as paclitaxel and podophyllotoxin. Zhao et al. [45] also discussed the relations between the endophytic fungi and their host plants and some available strategies for efficiently promoting the production of these bioactive compounds, as well as their potential applications in the future. However, further studies are warranted to investigate the active compounds from these endophytic fungi and explore the possibilities of the induction of these fungi by the host plant to produce beneficial active compounds. No mycoses insects were recorded, suggesting that the recorded mortality of the insect was as a result of the production of metabolites/antibiosis [9]. The effects of *H. lixii* and *B. bassiana* fungal extracts on insect larvae also did not show variation from the controls. This points to recorded effects being a result of compounds extracted from colonized plants, previously not in control plants and therefore induced by the fungi rather than compounds from the fungi itself. Methanol and dichloromethane solvents were both used to target both polar and non-polar compounds. However, Methanol extracts showed significant differences compared to controls in their effects against leafminer, but there was no significant difference in the effects of dichloromethane extracts compared to controls on leafminer. This suggests higher activity in methanol extracted compounds in contrast to dichloromethane extracted compounds. The LT_50_ values of the plant exposed to insects are higher than the ones that were not exposed to the insects. This difference could be a result of the feeding activity of the insects that stimulate the host plant to produce different metabolites that might be different from the ones induced through endophytes inoculation. However, further studies are warranted to elucidate this phenomenon.

## 4. Materials and Methods

### 4.1. Fungal Cultures, Suspensions Preparation and Viability Test

Two fungal isolates *Beauveria bassiana* G1LU3 and *Hypocrea lixii* F3ST1 were acquired from the International Centre of Insect Physiology and Ecology (*icipe*), Arthropod Germplasm Centre. The fungal isolates (*H. lixii* F3ST1 and *B. bassiana* G1LU3) were initially isolated from the aerial parts of maize plants. The isolates were cultured on potato dextrose agar (PDA) and then maintained at 25 ± 2 °C in complete darkness. Conidia were harvested by scraping the surface of the 2–3-week-old sporulating cultures with a sterile spatula as described by Akutse et al. [9]. The harvested conidia were mixed in 10 mL sterile distilled water containing 0.05% Triton X-100 and vortexed for 5 min to produce homogenous conidial suspensions. Conidial counts were made using a Neubauer Hemocytometer [46]. The conidial suspension was adjusted to 1 × 10^8^ conidia mL^−1^ through serial dilutions prior to inoculation of *P. vulgaris* seeds.

Spore viability was determined by plating 0.1 mL of 3 × 10^6^ conidia mL^−1^ onto 9 cm Petri dishes containing PDA. Three sterile microscope coverslips (2 × 2 cm) were placed on the top of the agar in each plate. Plates were incubated in complete darkness at 25 ± 2 °C and examined after 16–20 h. The percentage germination of conidia was determined from 100 randomly selected conidia on the surface area covered by each coverslip under the light microscope (400×) using the method described by Goettel and Inglis [46]. Conidia were deemed to have germinated when the length of the germ tube was at least twice the diameter of the conidium. Four replicates were used for each fungal isolate.

### 4.2. Seeds Inoculation and Endophytes Colonization Assessment

Inoculation was carried out by soaking *P. vulgaris* seeds (Brown Rose Coco) in conidial suspensions titrated at the concentration of 1 × 10^8^ conidia mL^−1^ for 2 h. Prior to inoculation, seeds were surface sterilized in 70% ethanol for 2 min followed by 1.5% sodium hypochlorite for 3 min, and rinsed three times with sterile distilled water. For the controls, sterilized seeds were soaked in sterile distilled water for 2 h as described by Gathage et al. [3]. The last rinse water was plated out to assess the effectiveness of the surface sterilization procedure [47]. Seeds were transferred into plastic pots (8 cm diameter × 7.5 cm high) containing the planting substrate (mixture of manure and soil 1:5). The planting substrate was sterilized in an autoclave for 2 h at 121 °C and allowed to cool for 72 h prior to planting [8]. Five seeds were sowed per pot and maintained at room temperature (25 ± 2 °C) and 60% relative humidity (RH). Pots were transferred immediately after germination to a screen house (2.8 m length × 1.8 m width × 2.2 m height) at 25 ± 2 °C, for 2 weeks. Seedlings were thinned to three per pot after germination and watered twice per day (morning and afternoon). No additional fertilizer was added to the substratum.

To determine the colonization of inoculated fungal isolates in *P. vulgaris*, plants were carefully removed from the pots two weeks after inoculation and washed with running tap water. Seedlings (ca. 30 cm in height) were cut into different sections (ca. 5 cm long): leaves, stems, and root sections. Five pieces of randomly selected leaf, stem, and root sections from each plant were surface sterilized as described above. The different plant parts were aseptically cut under a laminar flow hood into 1 × 1 cm pieces before placing the pieces, 4 cm apart on PDA plates amended with a 0.05% solution of antibiotic (streptomycin sulfate salt) [48]. Five pieces of each plant part were placed on a Petri dish, and the pieces with fungal outgrowth were counted out of 5, with results represented in percentages. Each treatment had 4 replicates (Petri dishes) with parts from a single plant. Plates were incubated at 25 ± 1 °C for 10 days, after which the presence of endophytes was determined. The last rinse water was also plated out to assess the effectiveness of the surface sterilization procedure as described above. The colonization of the different plant parts was recorded by counting the number of pieces of the different plant parts that showed the presence of inoculated fungal growth/mycelia according to Koch’s postulates [27]. Only the presence of endophytes that were inoculated were scored. Slides were prepared from the mother plates and used for morphological identification. Treatments were randomized in a complete block design (RCBD) and the experiment was replicated four times over time.

### 4.3. Insect Rearing and Treatments

Fall armyworm (FAW) *S. frugiperda* and leafminer (LMF) *L. huidobrensis* were obtained from the *icipe’s* Animal Rearing and Quarantine Unit (ARQU). The initial colonies originating from adult LMF and FAW that were collected from wild crucifers and maize crops, respectively, at the *icipe* campus (01°13.3′ S 36°53.8′ E, 1600 m a.s.l) and reared on beans and maize leaves, respectively, in plexiglass cages (50 cm × 50 cm × 45 cm) for 8 to 10 generations prior to experiments. LMF and FAW colonies were maintained at 27 ± 2 °C under a photoperiod of 12L:12D and relative humidity of approximately 50%. Two-week seedlings from the above treatments were used for secondary metabolites assessment. Two-week-old seedlings were randomly selected from each treatment and placed inside meshed cages (50 cm × 50 cm × 50 cm). These treatments included: endophyte-free plants (C—control plants), and endophyte colonized plants (F3—*H. lixii* colonized plants, G1—*B. bassiana* colonized plants). Plants from each treatment were placed in each cage and exposed to two-day-old, mated adult flies (30 individuals at sex ratio 1:2, male: female) for infestation. Treatments with leafminer therefore included (C2—control damaged plants, F32—*H. lixii* colonized damaged plants, G12—*B. bassiana* colonized damaged plants) and later also used for volatiles and secondary metabolites assessment.

### 4.4. Collection and Analysis of Volatiles

Volatiles released from the intact aerial parts of *P. vulgaris* plants inoculated with fungus, controls, and exposed to insects were collected by enclosing an intact plant in an air-tight plastic chamber and passing air through it (at a flow rate of 350 mL/minute) into adsorbent Super-Q traps. Talento timer-based volatile collection system was employed in capturing volatiles released at night (19:00–06:59). The Super-Q traps were eluted with 200 µL GC/GC-MS-grade dichloromethane (CH_2_Cl_2_) and the eluate was stored at −80 °C until used. Analysis of volatiles was carried out using a Hewlett-Packard (HP) 5890 Series II GC-MS equipped with an HP-1 column (30 m length × 0.25 mm inner diameter × 0.25 µm film thickness) with nitrogen as the carrier gas at 1 mL/min. Volatiles were analyzed in the splitless mode at an injector temperature of 280 °C and a split valve delay of 5 min. The oven temperature was held at 35 °C for 3 min, then programmed at 10 °C/minute to 280 °C and maintained at this temperature for 10 min [21]. For identification, the active compounds in the volatile extracts were analyzed by comparing their mass spectral data with those recorded in the Mass Spectral Library NIST/EPA/NIH 2005a.

### 4.5. Solvent Liquid Extractions

To assess larvicidal effects of compounds produced in the bean plants of the controls, endophyte treated and infested plants, solvent extraction using dichloromethane (CH_2_Cl_2_) and methanol (MeOH) was performed, to target the non-polar and moderately polar constituents. The above plant materials were collected and freeze-dried in liquid nitrogen. Dry samples were crushed using a pestle and mortar. Extraction of target compounds was processed by soaking the powdered material in solvents; MeOH and CH_2_Cl_2_ (5 g/mL) for 48 h with occasional stirring in the dark at laboratory temperature of 25 ± 2 °C. Whatman N° 1 filter papers were used to filter the crude extract. The solvents were evaporated, and the extract was concentrated under a vacuum using a rotary evaporator at 40 °C. The resulting dry residue of each treatment was expressed in mg.

### 4.6. Plant Extracts Bioassays

The leaf-dip bioassay described by Cahill et al. [49] was used to assess the biological effect of the extracts against the pests. These extracts were tested against the 2nd instar larvae of pea leafminer *L. huidobrensis* and the 1st instar larvae of fall armyworm *S. frugiperda* under laboratory conditions.

### 4.7. Statistical Analyses

Colonization data, percentage pupation, leafminer adult fly emergence, and FAW mortality data were analyzed with a generalized linear model (GLM) using binomial distribution and logit link function using the packages MASS and lme4 [50,51]. Where necessary, Abbott’s formula was applied to correct natural mortality [52]. The success rate (%) of fungal colonization of host plants parts were calculated using the following equation:

$$\mathrm{Colonization}\left(\%\right)=\frac{\mathrm{Number}\;\mathrm{of}\;\mathrm{pieces}\;\mathrm{exhibiting}\;\mathrm{fungal}\;\mathrm{outgrowth}}{\mathrm{Total}\;\mathrm{number}\;\mathrm{of}\;\mathrm{pieces}\;\mathrm{plated}\;\mathrm{out}}\times 100$$

All the analysis were performed using R (version 3.6.1) statistical software packages and all statistical results were considered significant at the confidence interval of 95% (*p* < 0.05) [53].

## 5. Conclusions

*Phaseolus vulgaris* was successfully colonized by *H. lixii* F3ST1 and *B. bassiana* G1LU3 through seed inoculation. Qualitative differences in volatile emissions and liquid extracts between the endophyte colonized plants and non-colonized plants elucidate a possible systemic infection mechanism of the endophytically colonized host plant against the pest. Identified volatile compounds were largely terpenes with previous histories as defense compounds for various plants against both microbial pathogens and herbivorous insects. The crude extract was tested and found to have negative effects on the larvae of both the LMF and FAW. Liquid extracts of the inoculated plants significantly reduced the pupation and emergence of LMF and the survival of FAW larvae in vitro. This warrants further research on the effects of the individual pure compounds that have been identified against the pests in addition to the dose-response bioassays.

## Figures and Tables

**Table 1:** Volatiles characterized from *Phaseolus vulgaris* (*n* = 3).

Compounds	Control Plants	LMF Damaged Control Plants	*H. lixii* Inoculated Plants	LMF Damaged *H. lixii* Inoculated Plants	*B. bassiana* Inoculated Plants	LMF Damaged *B. bassiana* Inoculated Plants
	Retention time (Area percentage)					
*m*-Cresol (**1**)	13.29 (35.63)	-	12.60 (72.92)	-	-	-
*p*-Cresol (**2**)	13.63 (64.36)	-	13.22 (13.28)	13.20 (6.50)	12.84 (0.74)	-
β-Phellandrene (**3**)	-	11.86 (1.21)	-	-	-	-
α-Terpinene (**4**)	-	12.35 (1.41)	-	-	-	12.33 (0.94)
*cis*-Sabinene hydrate (**5**)	-	12.51 (4.77)	-	-	-	13.02 (3.73)
*trans*-Sabinene hydrate (**6**)	-	13.04 (4.66)	-	-	-	-
Camphor (**7**)	-	13.81 (1.56)	-	-	-	13.81 (0.99
Terpinen-4-ol (**8**)	-	14.34 (28.68)	-	-	-	14.32 (16.68)
(*E*)-Caryophyllene (**9**)	-	17.84 (2.28)	-	18.17 (1.23)	17.81 (2.08)	-
Benzaldehyde, dimethyl acetal (**10**)	-	13.25 (21.83)	-	14.48 (0.47)	13.20 (11.88)	13.22 (16.51)
Heneicosane (**11**)	-	15.87 (2.02)	-	17.14 (1.49)	21.02 (1.30)	19.90 (4.53)
Butylated hydroxytoluene (**12**)	-	18.93 (6.38)	-	19.85 (3.32)	18.89 (5.38)	18.91 (4.64)
*cis*-1,1,3,5-Tetramethyl cyclohexane (**13**)	-	-	9.46 (0.89)	-	-	-
Phenol (**14**)	-	-	11.03 (1.47)	-	-	-
Benzyl alcohol (**15**)	-	-	11.97 (6.88)	8.23 (0.09)	-	-
4-Methyloctane (**16**)	-	-	-	8.23 (0.09)	-	-
3-Methylanisole (**17**)	-	-	-	11.93 (0.45)	-	-
(*Z*)-β-ocimene (**18**)	-	-	-	11.92 (0.44)	11.95 (0.31)	-
(*E*)-β-ocimene (**19**)	-	-	-	12.13 (1.97)	12.13 (1.41)	-
Naphthalene (**20**)	-	-	-	14.50 (0.37)	14.50 (0.37)	-
Methyl salicylate (**21**)	-	-	-	14.68 (3.14)	14.68 (4.47)	-
Heptadecane (**22**)	-	-	-	15.89 (0.27)	-	-
6-Propyl-tridecane (**23**)	-	-	-	16.07 (0.44)	-	-
Propyl butanoate (**24**)	-	-	-	16.68 (0.34)	-	-
Tridecane (**25**)	-	-	-	17.39 (1.06)	-	-
α-Cedrene (**26**)	-	-	-	17.73 (0.56)	17.75 (0.57)	17.77 (1.50)
Octadecane (**27**)	-	-	-	17.82 (1.65)	-	-
Tetradecane (**28**)	-	-	-	18.89 (4.86)	17.42 (0.80)	-
Dibutyl phthalate (**29**)	-	-	-	23.96 (1.17)	24.02 (1.33)	-
1-Methoxy-3-methylbenzene (**17**)	-	-	-	-	11.66 (0.40)	-
(*E*)-γ-Bisabolene (**30**)	-	-	-	-	18.81 (4.04)	-
4,8,12-Trimethyl-1,3*E*,7*E*,11-tridecatetraene (**31**)	-	-	-	-	19.68 (4.33)	-
Sulfurous acid, pentyl undecyl ester (**32**)	-	-	-	-	19.86 (3.37)	-
Benzaldehyde (**33**)	-	-	-	-	-	10.70 (0.61)
5,7-Dimethyl undecane (**34**)	-	-	-	-	-	15.85 (1.45)
2-Methyl-2-ethyl-3-hydroxyhexyl propanoate (**35**)	-	-	-	-	-	17.24 (0.77)

(-): not detected.

**Table 2:** Median lethal time (LT_50_) 7 days post-treatment of 1st instar FAW larvae dipped into fungal and plant extracts.

Extracts	Treatments	LT_50_ (Days) (95% FL)
Fungal extracts	*H. lixii* F3ST1	6.24 (6.41–6.07)
	*B. bassiana* G1LU3	6.73 (6.95–6.51)
Plant extracts	C1M	9.36 (9.83–8.89)
	C2M	16.47 (19.73–13.21)
	C1D	17.06 (19.43–14.69)
	C2D	10.79 (11.56–10.02)
	F31M	4.50 (4.62–4.38)
	F32M	5.58 (5.72–5.44)
	F31D	6.85 (6.89–6.27)
	F32D	8.70 (9.07–8.33)
	G11M	7.87 (8.25–7.49)
	G12M	4.42 (4.59–4.25)
	G11D	4.72 (4.83–4.61)
	G12D	5.76 (5.91–5.61)

## Data Availability

Not applicable.

## Supplementary Materials

The following are available online. GC-MS profiles of volatiles characterized from *Phaseolus vulgaris* can be found in this section.

## Sample Availability

Samples of the compounds are not available from the authors.
