# Supplementary material for: Mechanism of Action of Endophytic Fungi Hypocrea lixii and Beauveria bassiana in Phaseolus vulgaris as Biopesticides against Pea Leafminer and Fall Armyworm
Source: Molecules. 2021 Sep 20;26(18):5694. doi: 10.3390/molecules26185694 (PMC8471441; doi:10.3390/molecules26185694)
Supplement: Supplementary file 1 [file molecules-26-05694-s001.zip › molecules-1327903-supplementary.pdf]

# SUPPLEMENTARY MATERIALS

# Mechanism of Action of Endophytic Fungi *Hypocrea lixii* and *Beauveria bassiana* in *Phaseolus vulgaris* as Biopesticides against Pea Leafminer and Fall Armyworm

**Olivia Ngeno Chebet [1,2], Leonidah Kerubo Omosa [1,*], Sevgan Subramanian [2], Vaderament-A Nchiozem-Ngnitedem [1], John Onyari Mmari [1] and Komivi Senyo Akutse [2,*]**

1 Department of Chemistry, University of Nairobi, P.O. Box 30197, Nairobi 00100, Kenya; ngenoolivia@gmail.com (O.N.C.); n.vaderamentalexe@gmail.com (V.-A.N.-N.); jonyari@uonbi.ac.ke (J.O.M.)

2 International Centre of Insect Physiology and Ecology (*icipe*), P.O. Box 30772, Nairobi 00100, Kenya; ssubramania@icipe.org

* Correspondence: lkerubo@uonbi.ac.ke (L.K.O.); kakutse@icipe.org (K.S.A.); Tel: +254-72-179-7175 (L.K.O.); +254-79-971-6741 (K.S.A.); Fax: +254-20-446-138 (L.K.O.); +254-20-8632-001/2 (K.S.A.)

## Table of Contents

Figure S1: Mass spectrum of *m*-Cresol (**1**) .......... 3

Figure S2: Mass spectrum of *p*-Cresol (**2**) .......... 3

Figure S3: Mass spectrum of β-Phellandrene (**3**) .......... 4

Figure S4: Mass spectrum of α-Terpinene (**4**) .......... 4

Figure S5: Mass spectrum of *cis*-Sabinene hydrate (**5**) .......... 5

Figure S6: Mass spectrum of *trans*-Sabinene hydrate (**6**) .......... 5

Figure S7: Mass spectrum of Camphor (**7**) .......... 6

Figure S8: Mass spectrum of Terpinen-4-ol (**8**) .......... 6

Figure S9: Mass spectrum of (*E*)-Caryophyllene (**9**) .......... 7

Figure S10: Mass spectrum of Benzaldehyde, dimethyl acetal (**10**) .......... 7

Figure S11: Mass spectrum of Heneicosane (**11**) .......... 8

Figure S12: Mass spectrum of Butylated hydroxytoluene (**12**) .......... 8

Figure S13: Mass spectrum of 3,6,6-Trimethylcyclohex-2-enol (**13**) .......... 9

Figure S14: Mass spectrum of Phenol (**14**) ........ 9
Figure S15: Mass spectrum of Benzylalcohol (**15**) ........ 10
Figure S16: Mass spectrum of 4-Methyloctane (**16**) ........ 10

Figure S17: Mass spectrum of 3-Methylanisole (**17**) ........ 11
Figure S18: Mass spectrum of (Z)-β-Ocimene (**18**) ........ 11
Figure S19: Mass spectrum of (*E*)-β-Ocimene (**19**) ........ 12
Figure S20: Mass spectrum of Naphthalene (**20**) ........ 12
Figure S21: Mass spectrum of Methylsalicylate (**21**) ........ 13
Figure S22: Mass spectrum of Heptadecane (**22**) ........ 13
Figure S23: Mass spectrum of 6-Propyl-tridecane (**23**) ........ 14
Figure S24: Mass spectrum of Propyl butanoate (**24**) ........ 14
Figure S25: Mass spectrum of Tridecane (**25**) ........ 15
Figure S26: Mass spectrum of α-Cedrene (**26**) ........ 15
Figure S27: Mass spectrum of Octadecane (**27**) ........ 16
Figure S28: Mass spectrum of Tetradecane (**28**) ........ 16
Figure S29: Mass spectrum of Dibutyl phthalate (**29**) ........ 17
Figure S30: Mass spectrum of (*E*)-γ-Bisabolene (**30**) ........ 17
Figure S31: Mass spectrum of 4,8,12-Trimethyl-1,3*E*,7*E*,11-tridecatetraene (**31**) ........ 18
Figure S32: Mass spectrum of Sulfurous acid, pentyl undecyl ester (**32**) ........ 18
Figure S33: Mass spectrum of Benzaldehyde (**33**) ........ 19
Figure S34: Mass spectrum of 5,7-Dimethylundecane (**34**) ........ 19
Figure S35: Mass spectrum of 2-Methyl-2-ethyl-3-hydroxyhexylpropanoate (**35**) ........ 20

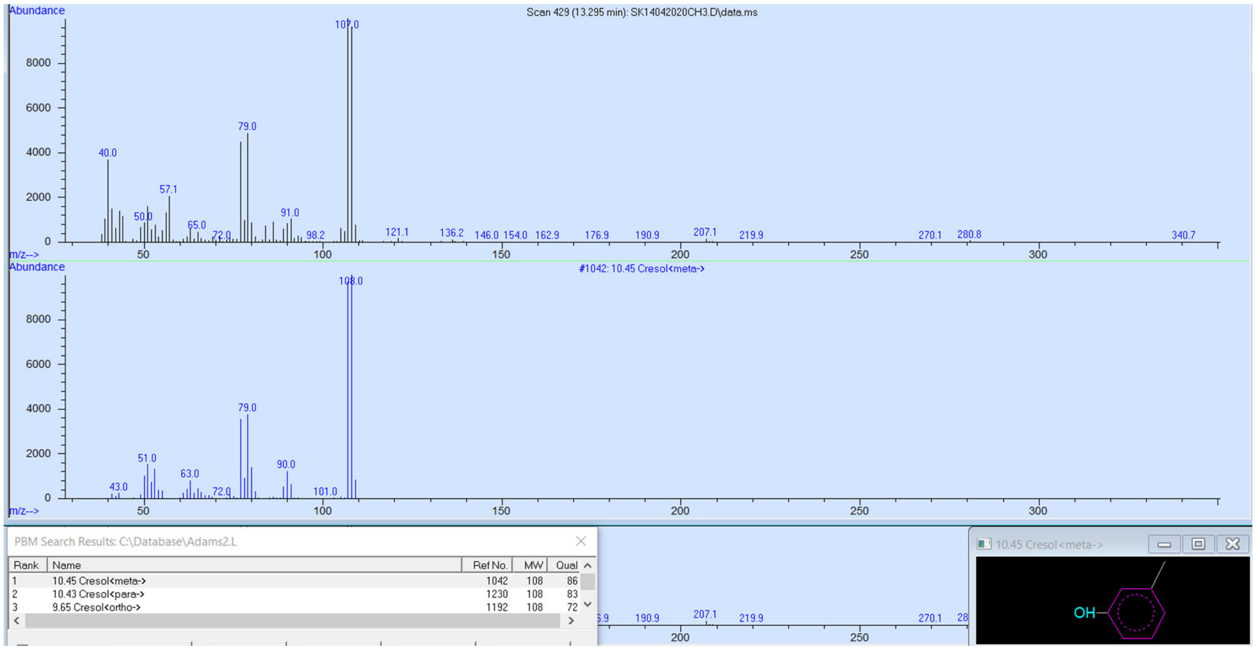


**Figure S1.** Mass spectrum of *m*-Cresol (**1**)

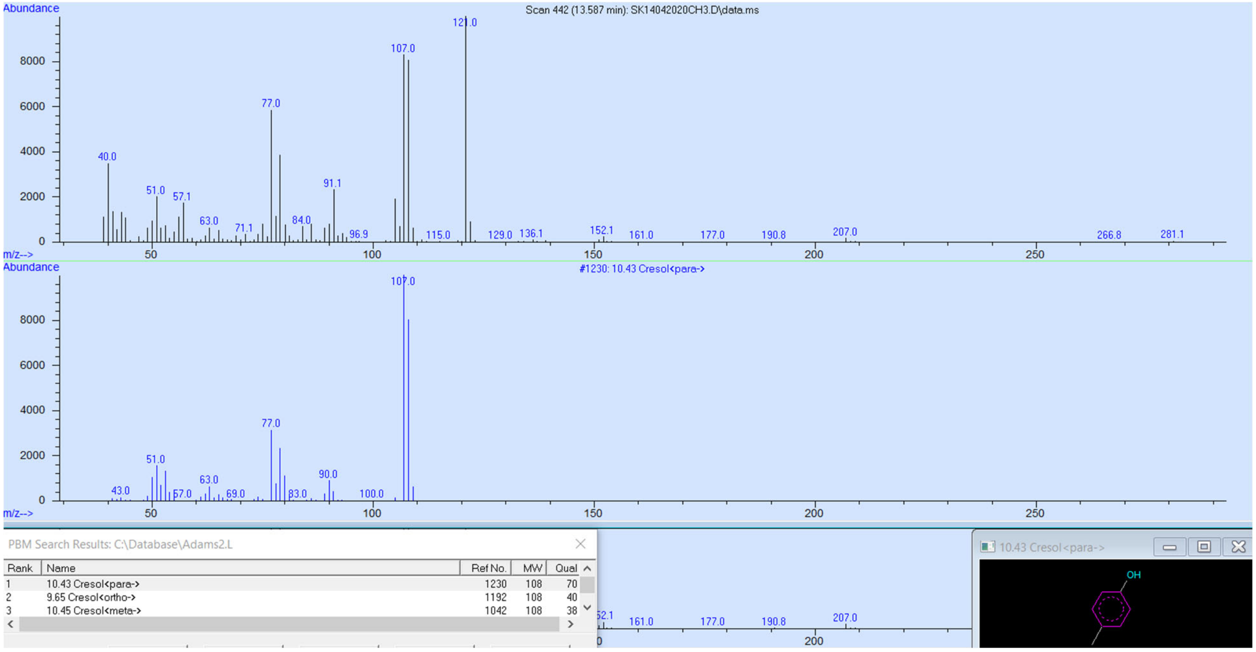


**Figure S2.** Mass spectrum of *p*-Cresol (**2**)

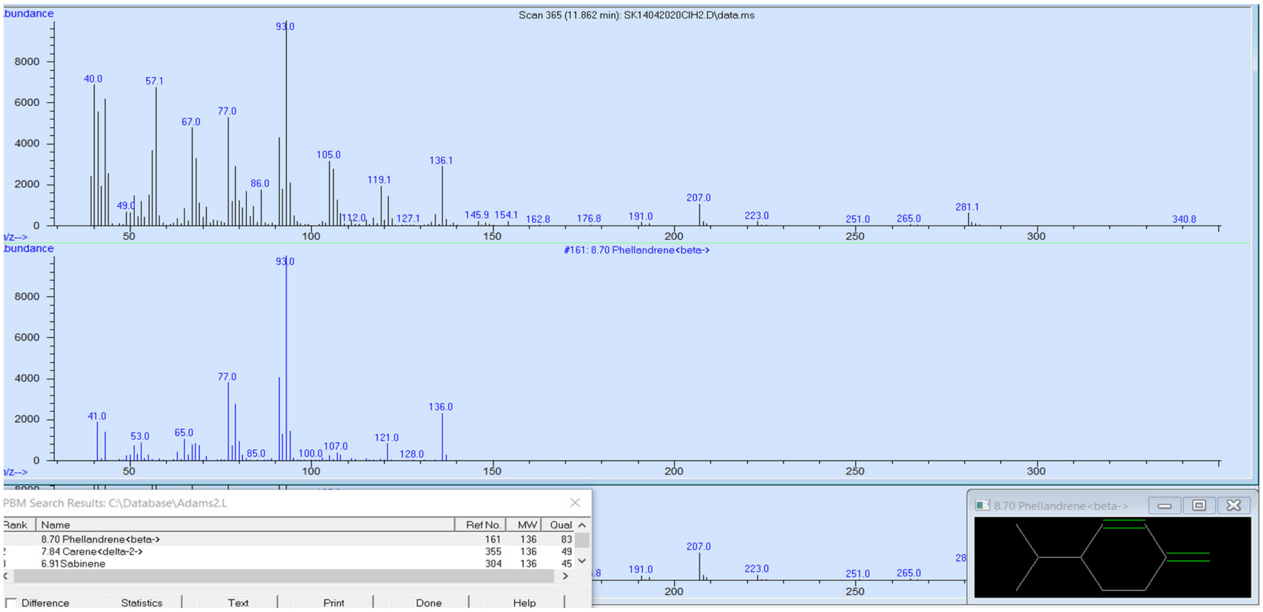


**Figure S3.** Mass spectrum of β-Phellandrene (**3**)

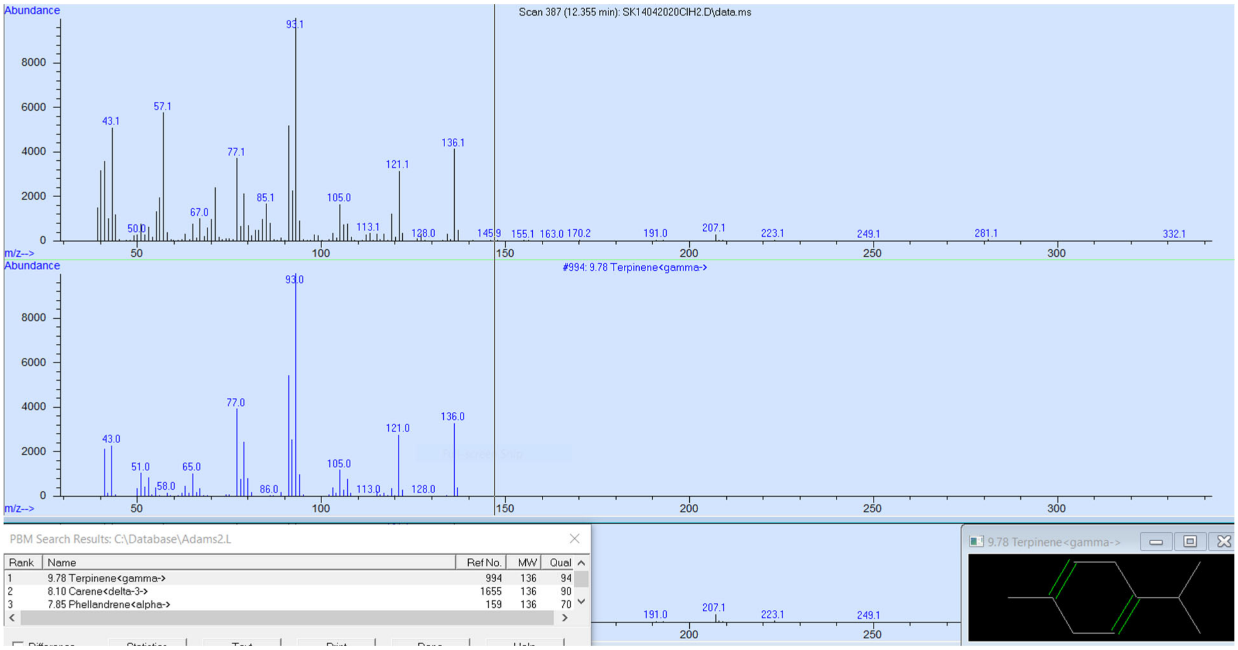


**Figure S4.** Mass spectrum of α-Terpinene (**4**)

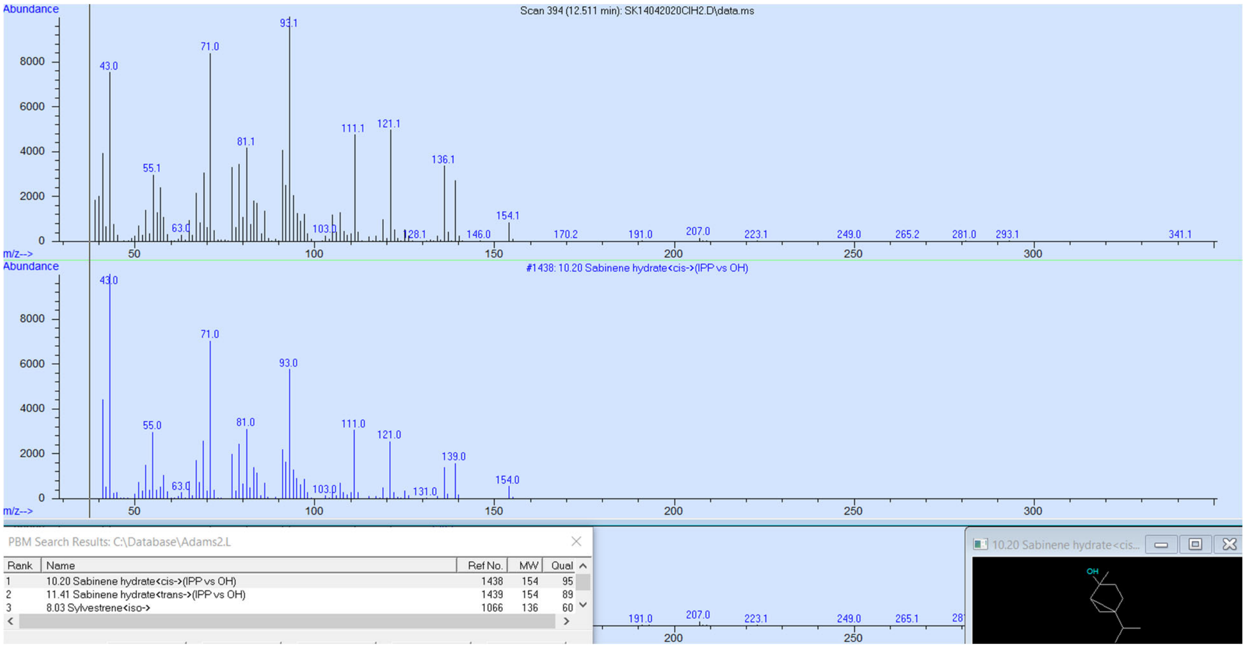


**Figure S5.** Mass spectrum of *cis*-Sabinene hydrate (**5**)

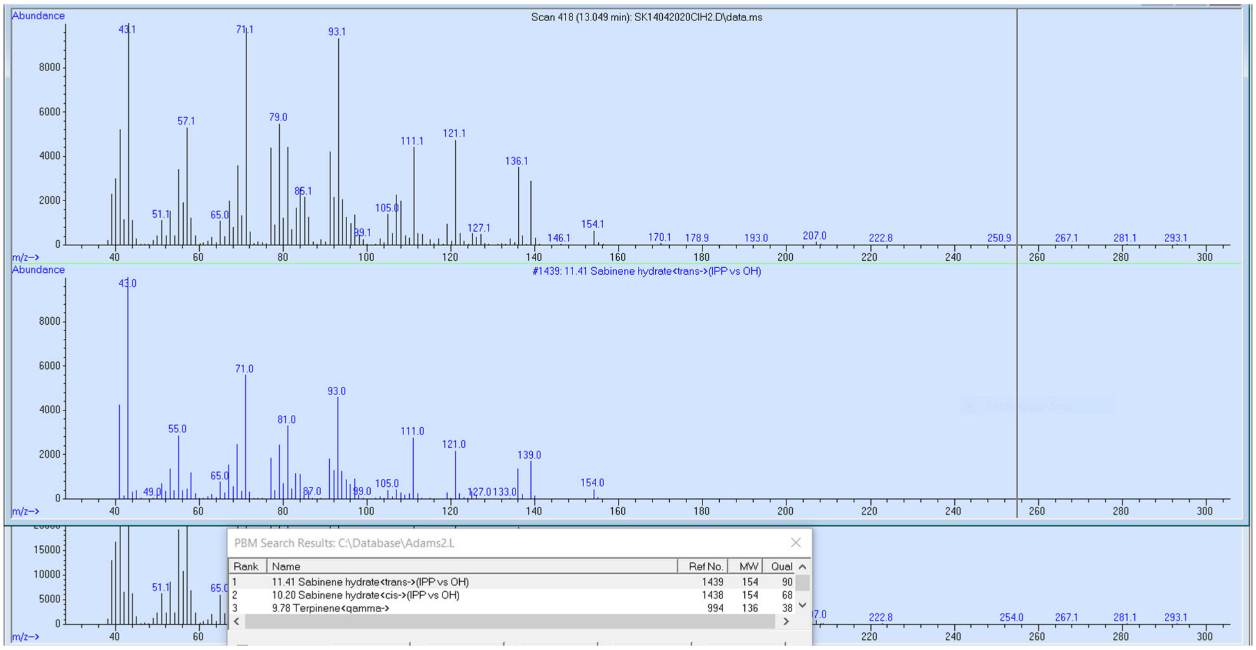


**Figure S6.** Mass spectrum of *trans*-Sabinene hydrate (**6**)

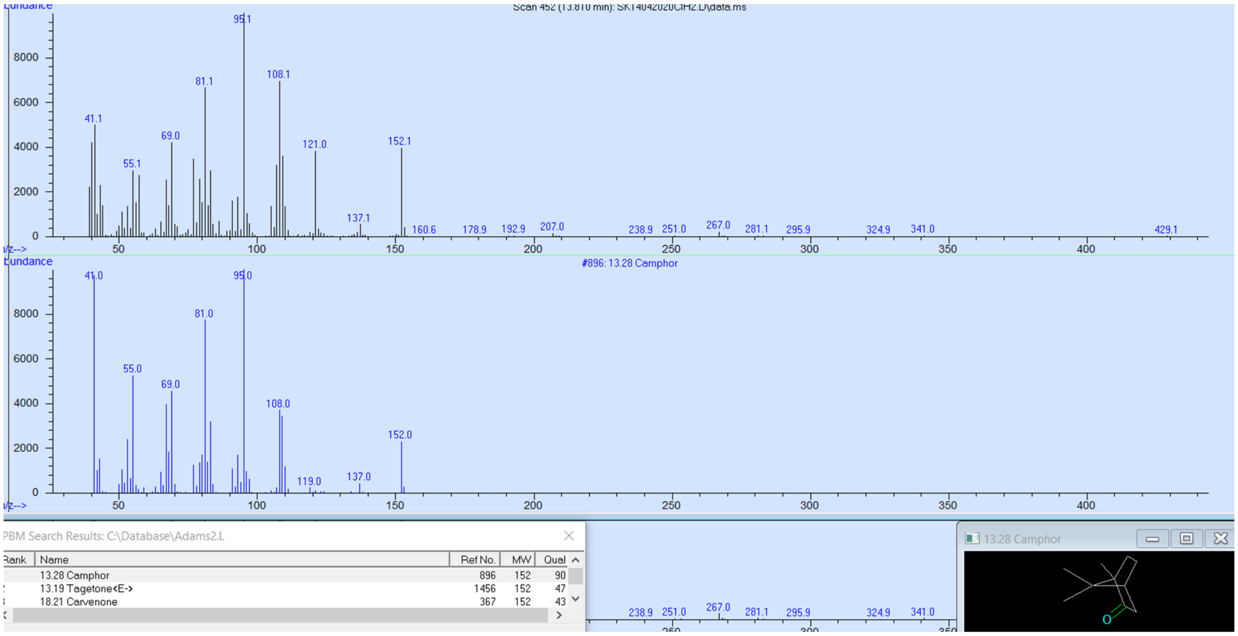


**Figure S7.** Mass spectrum of Camphor (**7**)

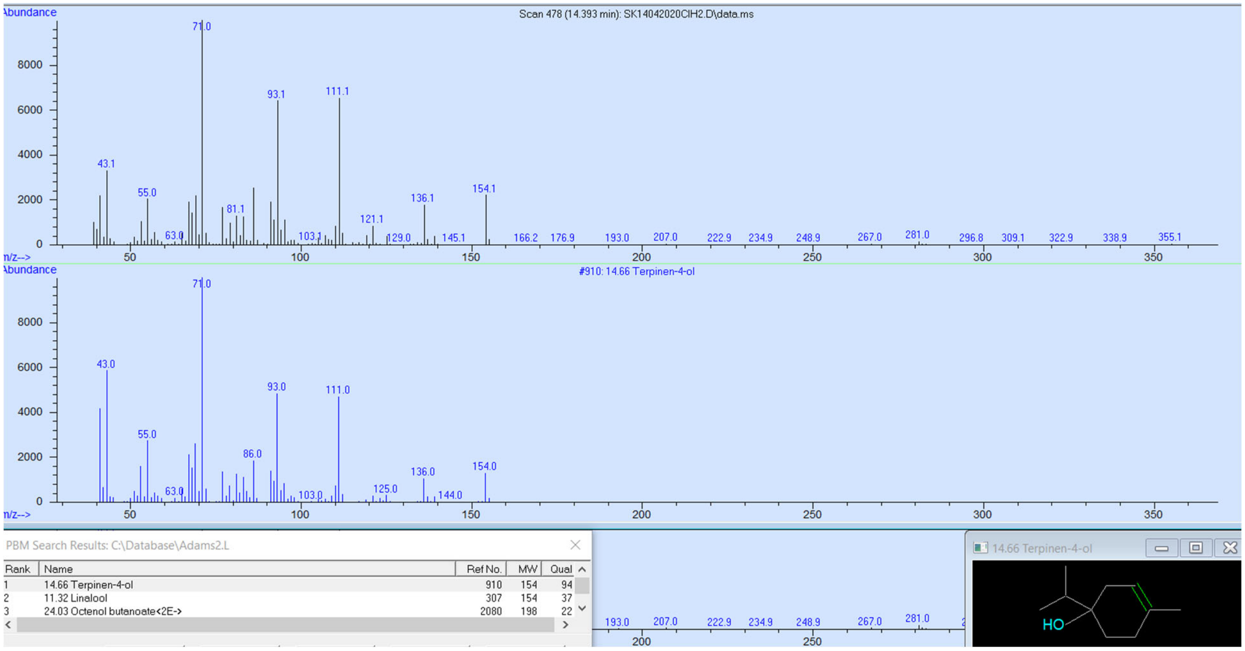


**Figure S8.** Mass spectrum of Terpinen-4-ol (**8**)

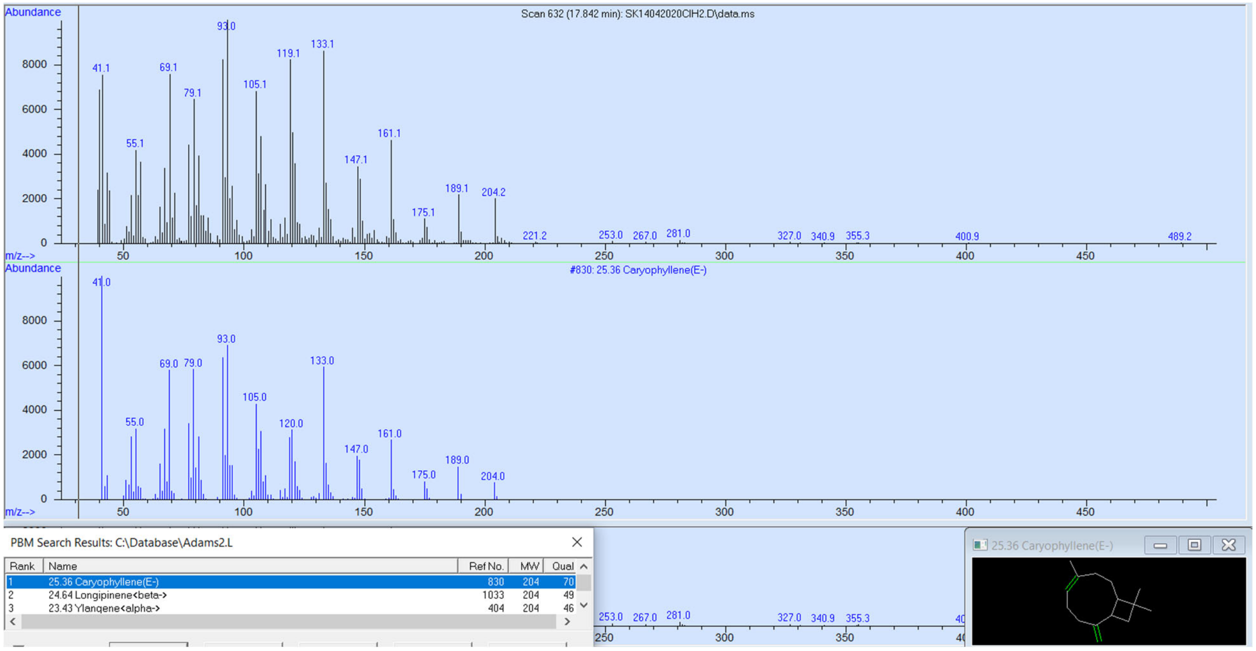


**Figure S9.** Mass spectrum of (*E*)-Caryophyllene (**9**)

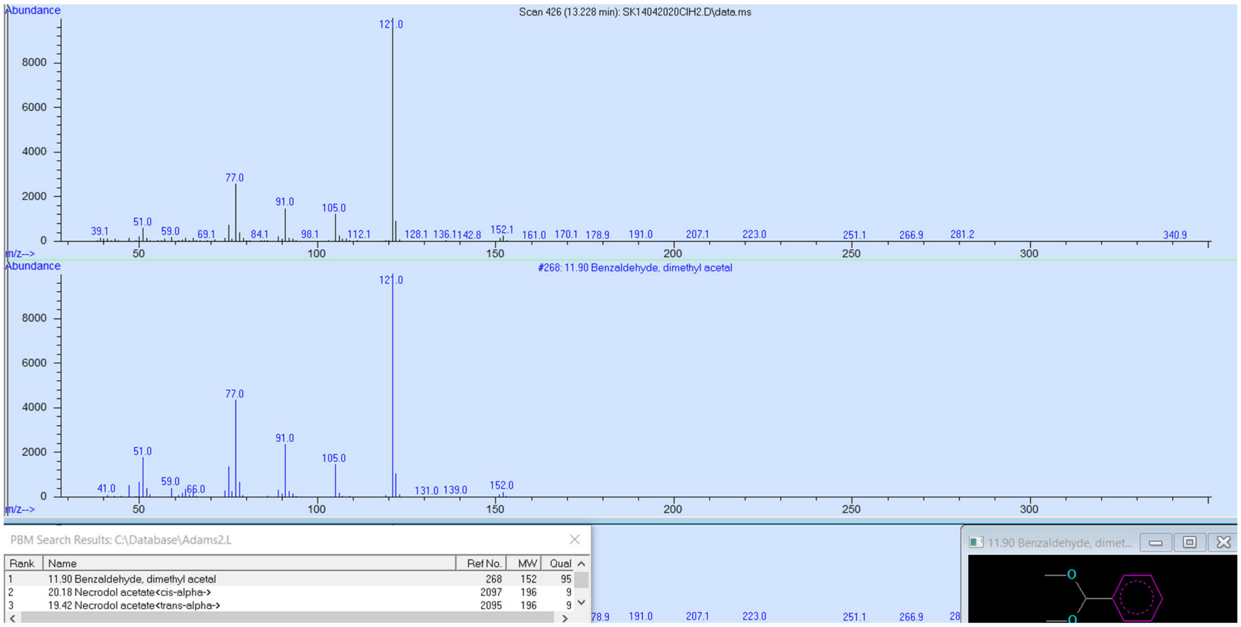


**Figure S10**. Mass spectrum of Benzaldehyde, dimethyl acetal (**10**)

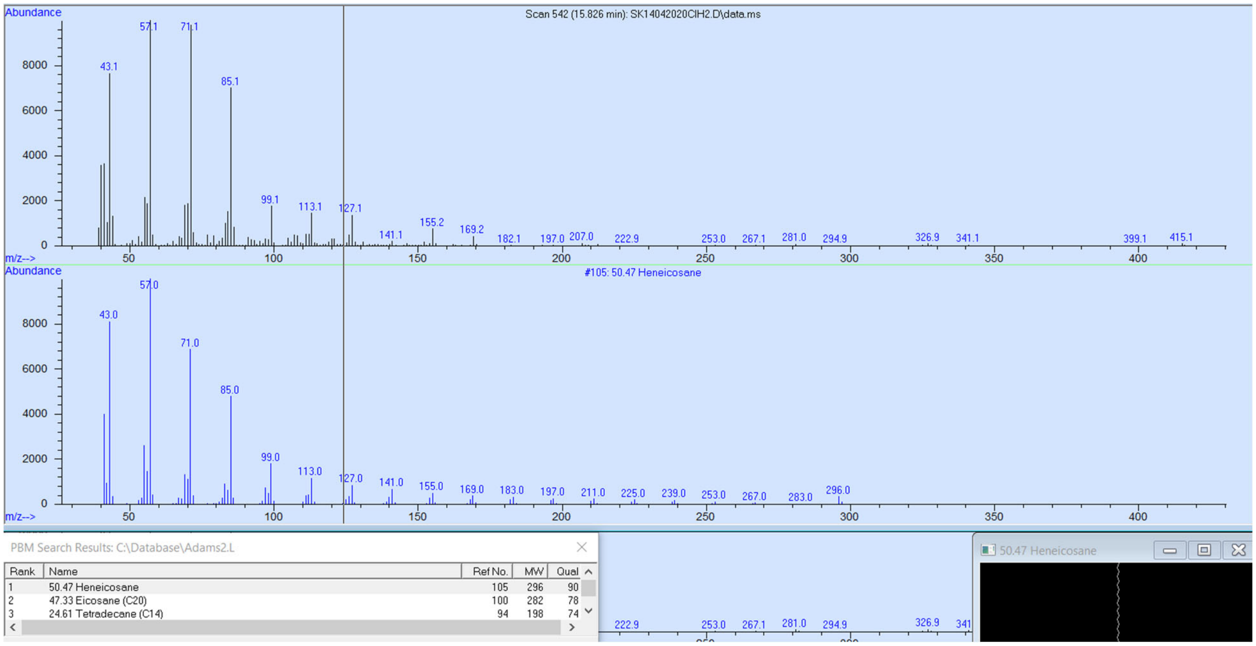


**Figure S11.** Mass spectrum of Heneicosane (**11**)

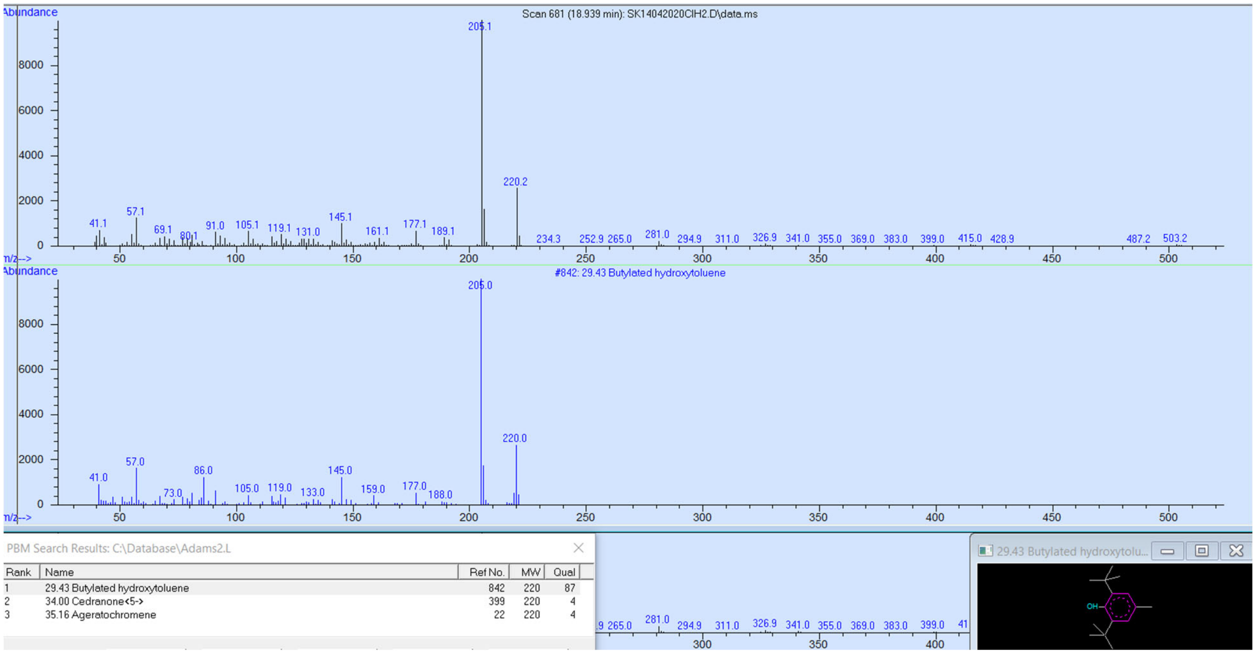


**Figure S12.** Mass spectrum of Butylated hydroxytoluene (**12**)

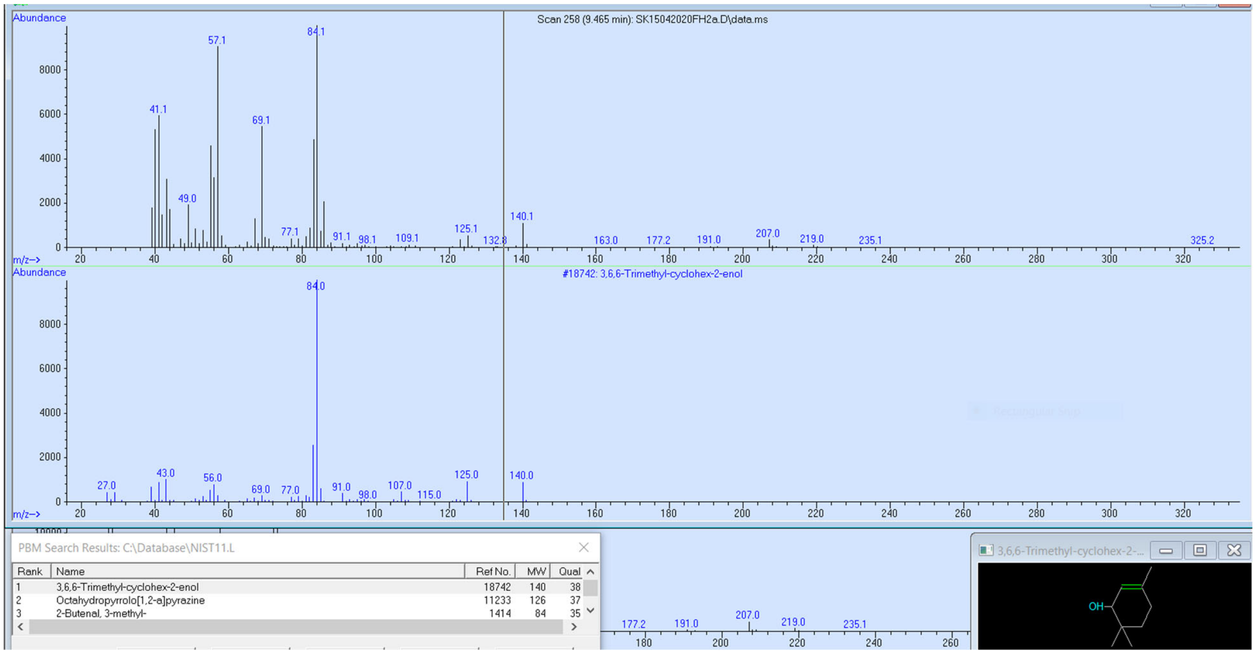


**Figure S13.** Mass spectrum of 3,6,6-Trimethylcyclohex-2-enol (**13**)

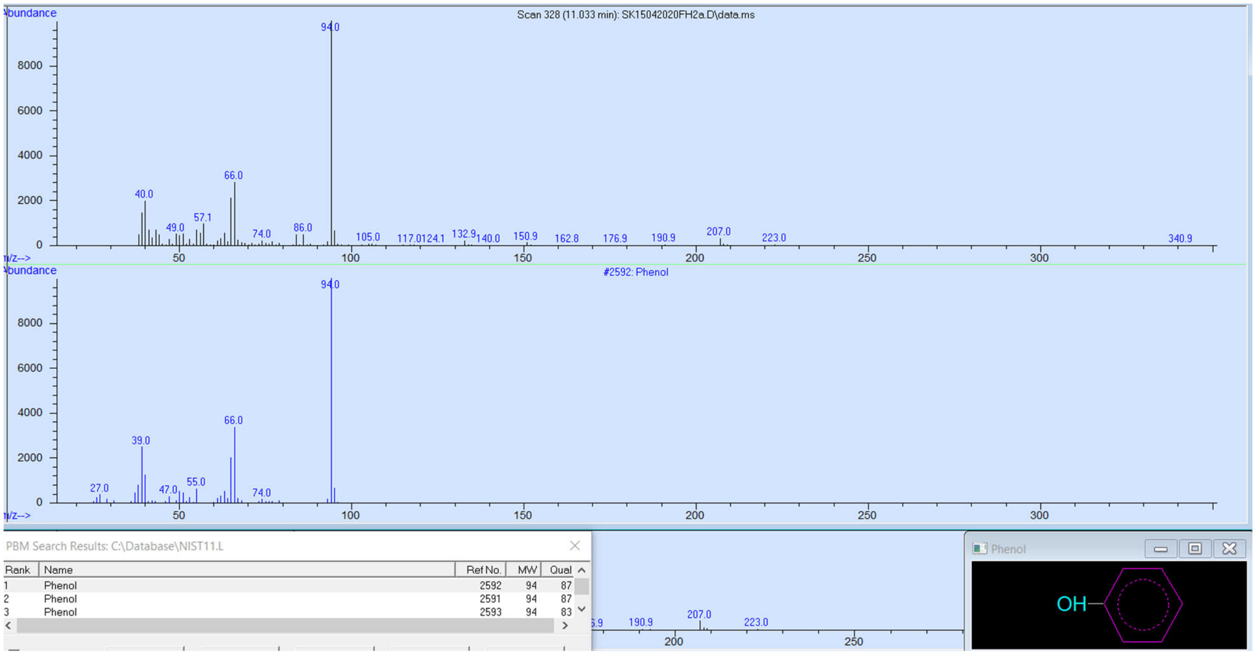


**Figure S14**. Mass spectrum of Phenol (**14**)

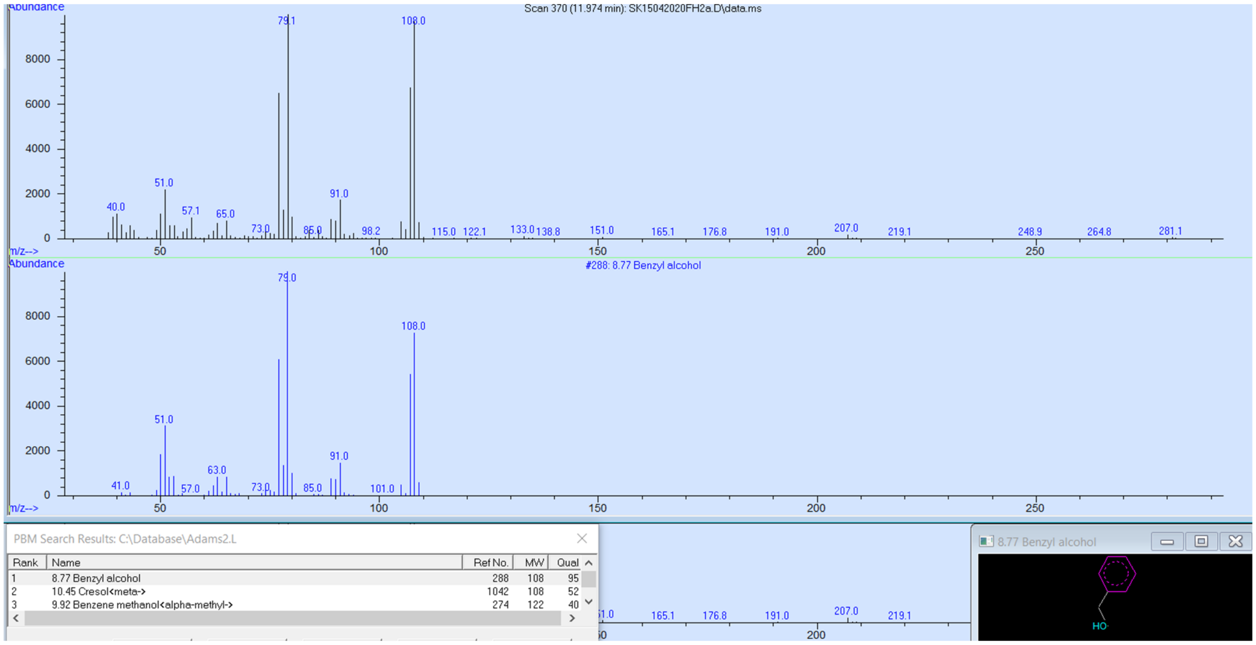


**Figure S15.** Mass spectrum of Benzylalcohol (**15**)

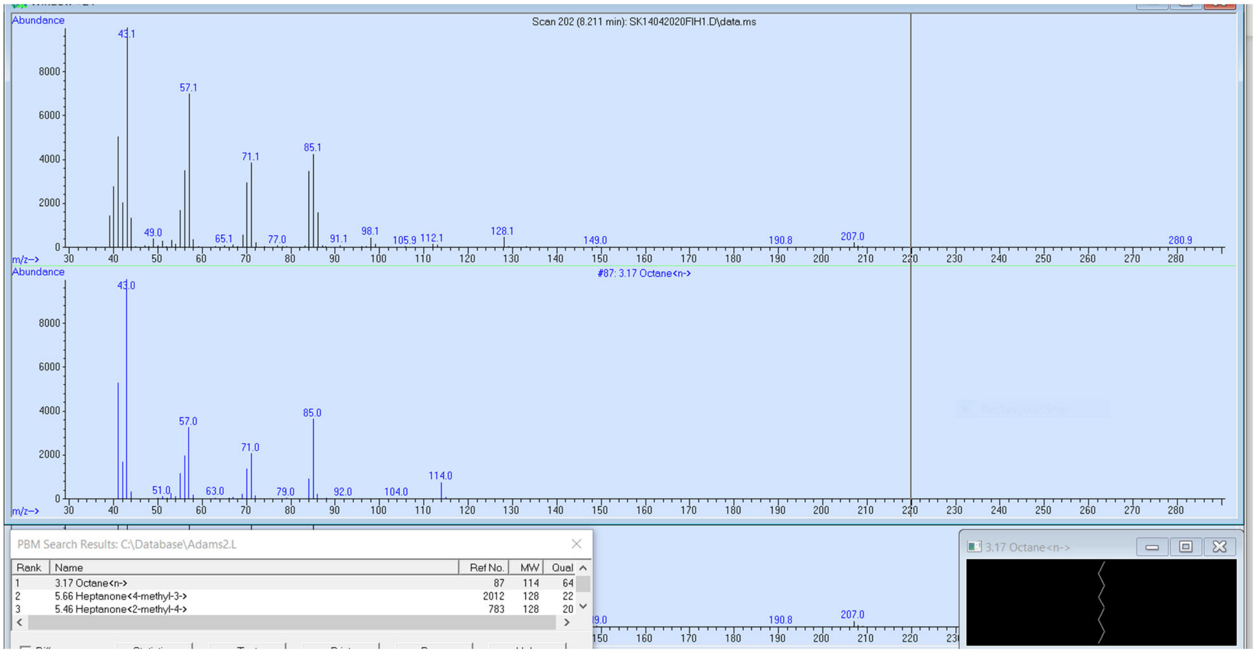


**Figure S16.** Mass spectrum of 4-Methyloctane (**16**)

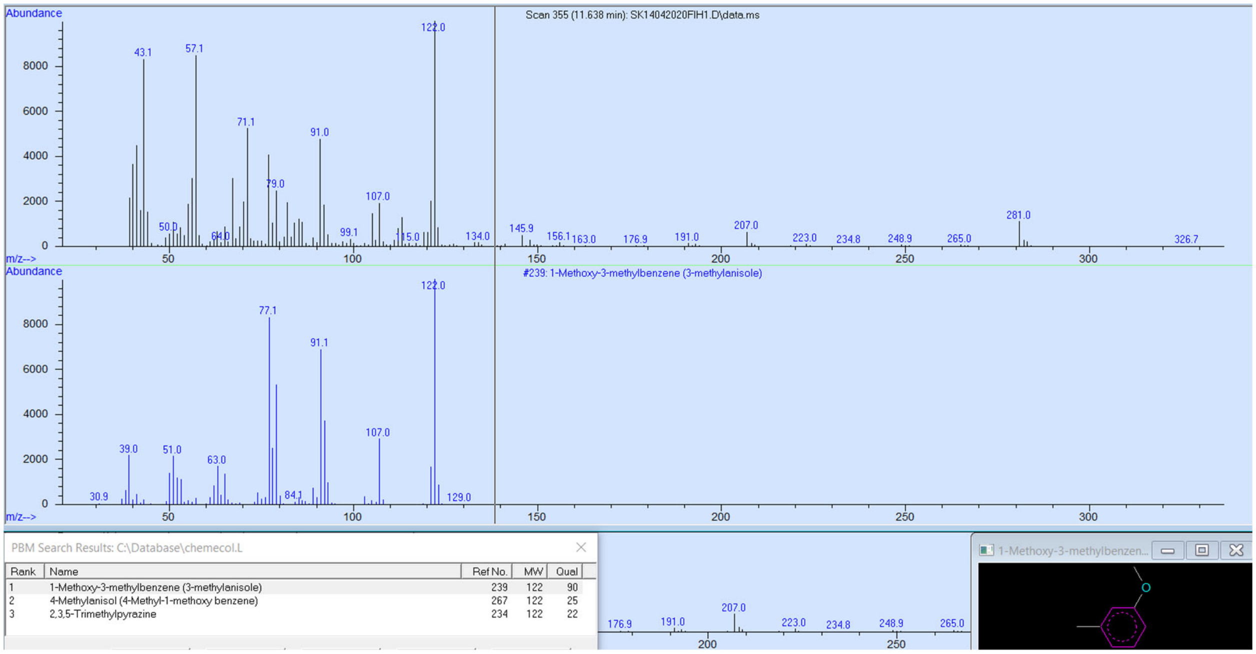


**Figure S17.** Mass spectrum of 3-Methylanisole (**17**)

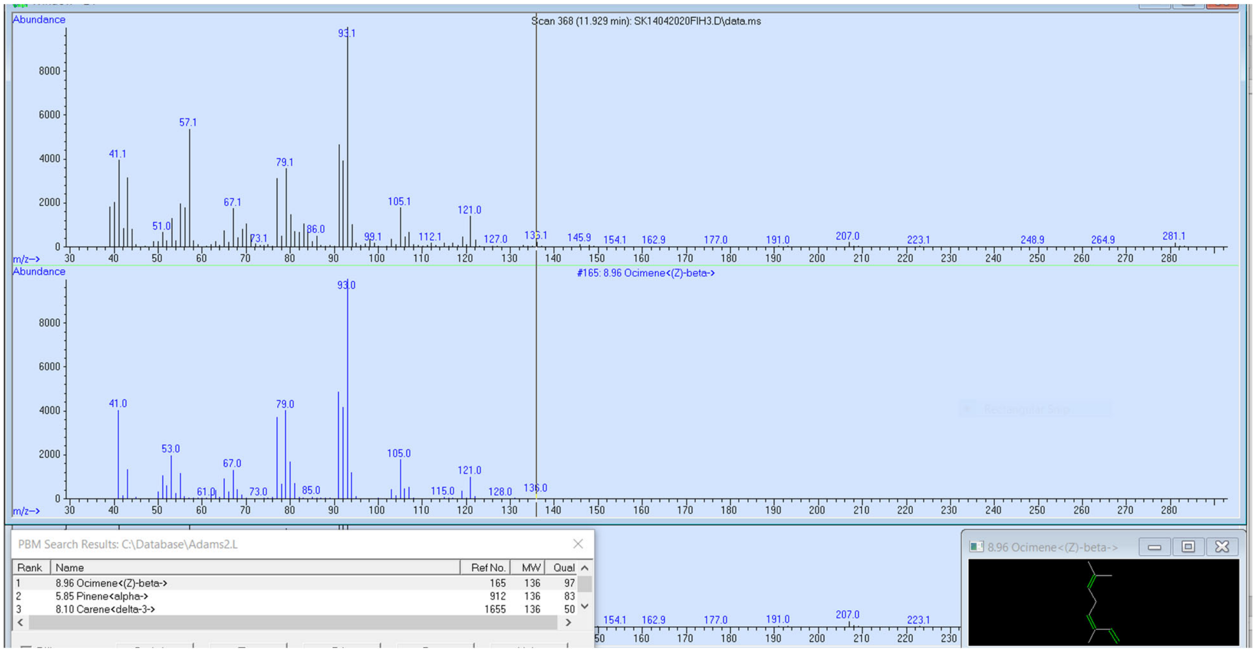


**Figure S18.** Mass spectrum of (*Z*)-β-Ocimene (**18**)

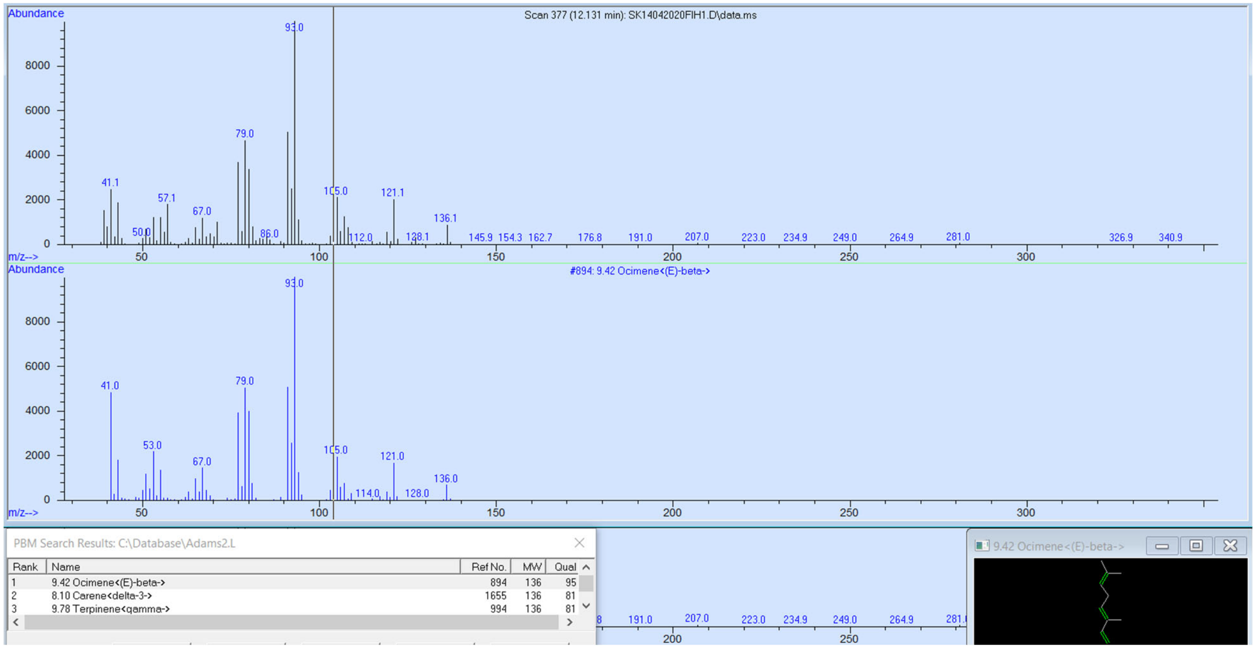


Figure S19. Mass spectrum of (*E*)-β-Ocimene (**19**)

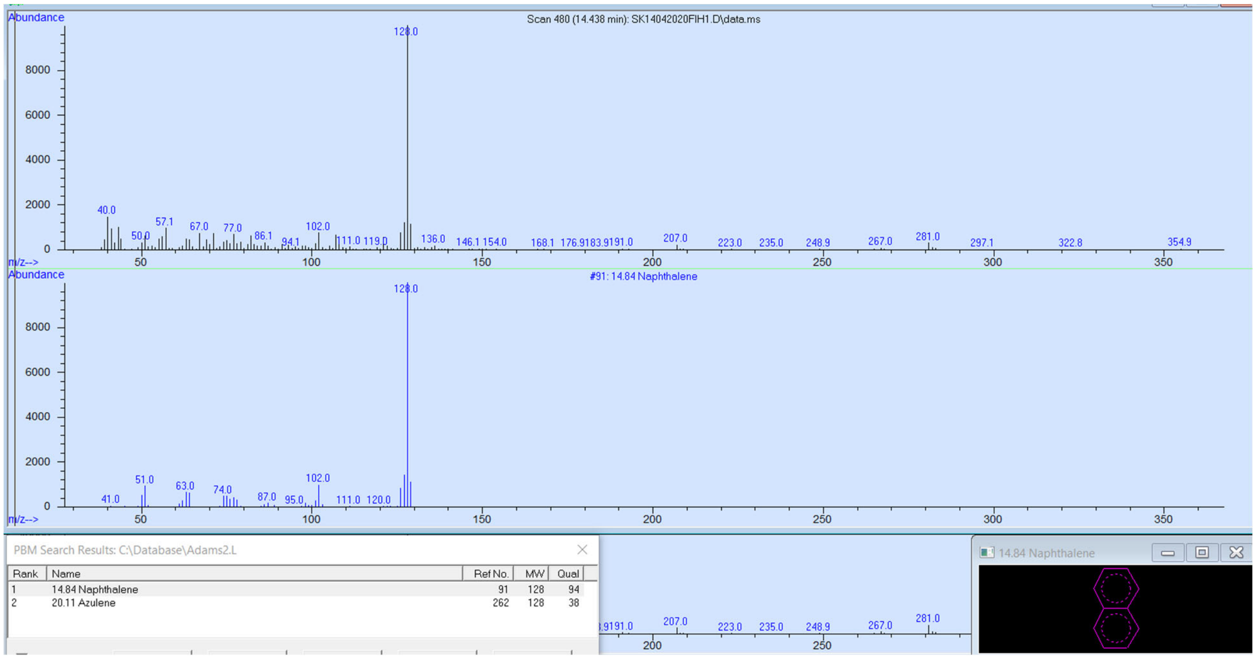


**Figure S20.** Mass spectrum of Naphthalene (**20**)

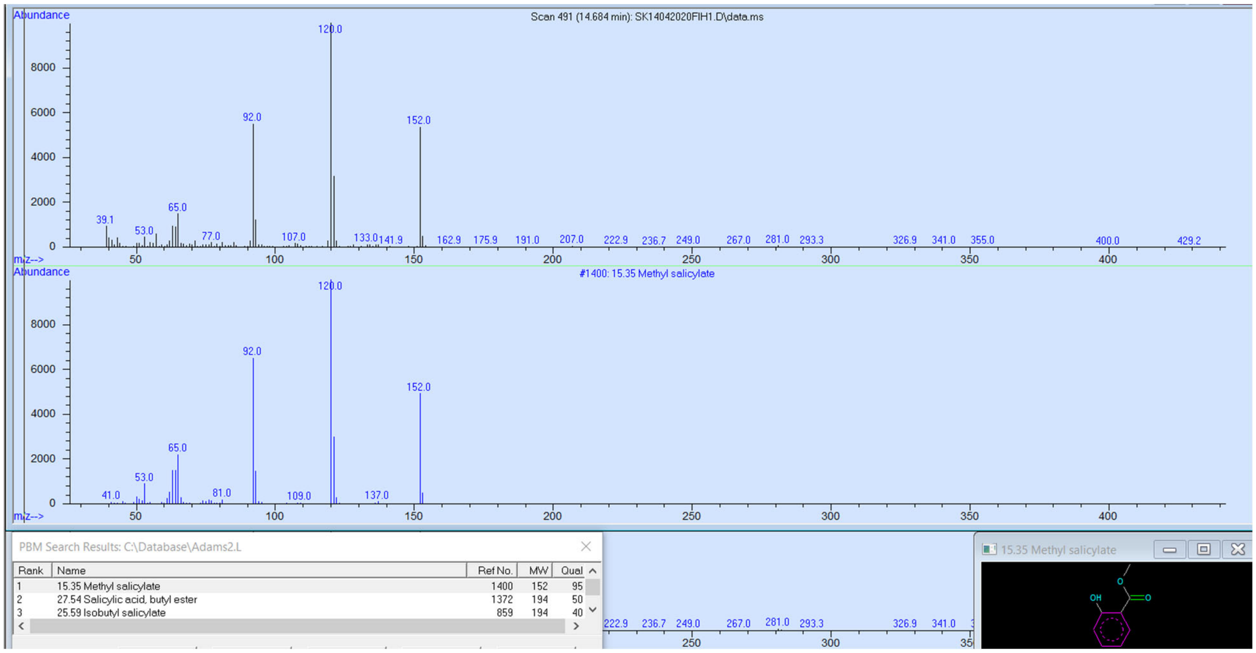

**Figure S21.** Mass spectrum of Methylsalicylate (**21**)

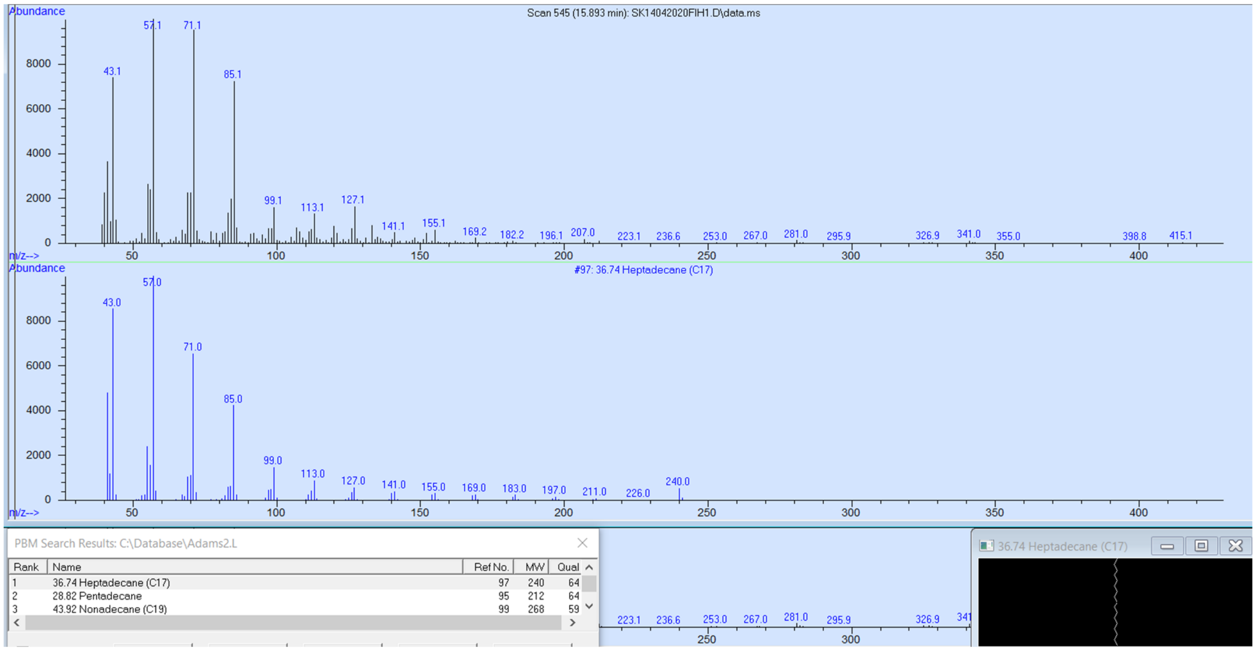

**Figure S22**. Mass spectrum of Heptadecane (**22**)

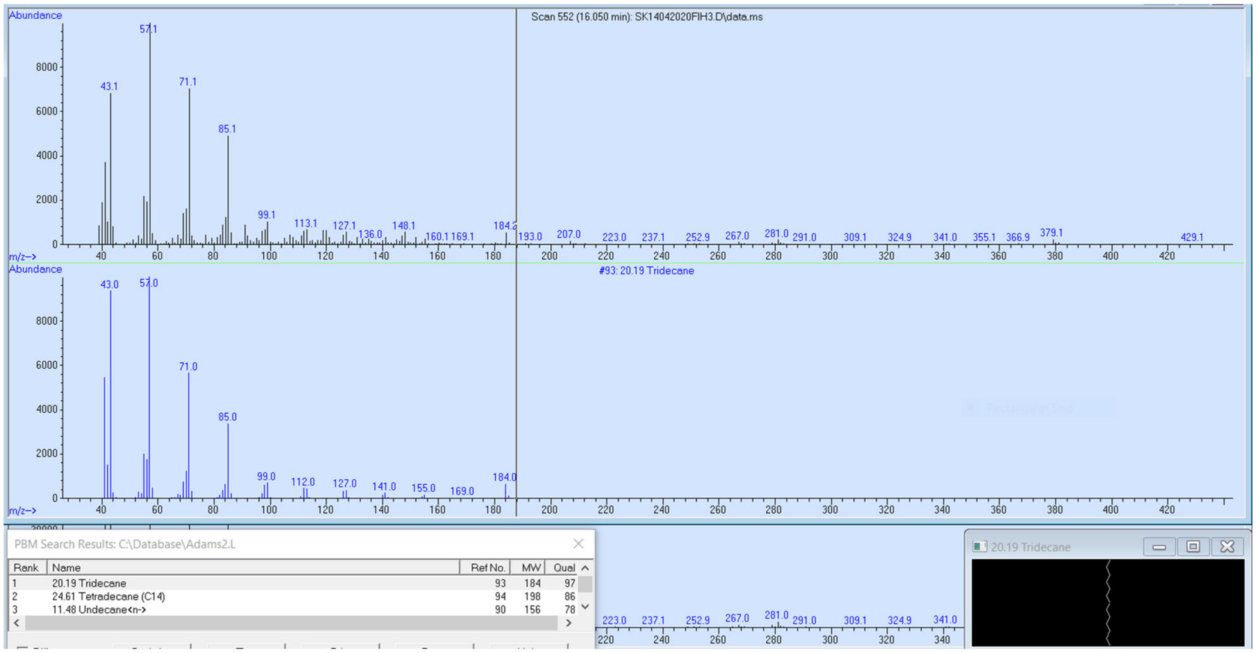


**Figure S23.** Mass spectrum of 6-Propyl-tridecane (**23**)

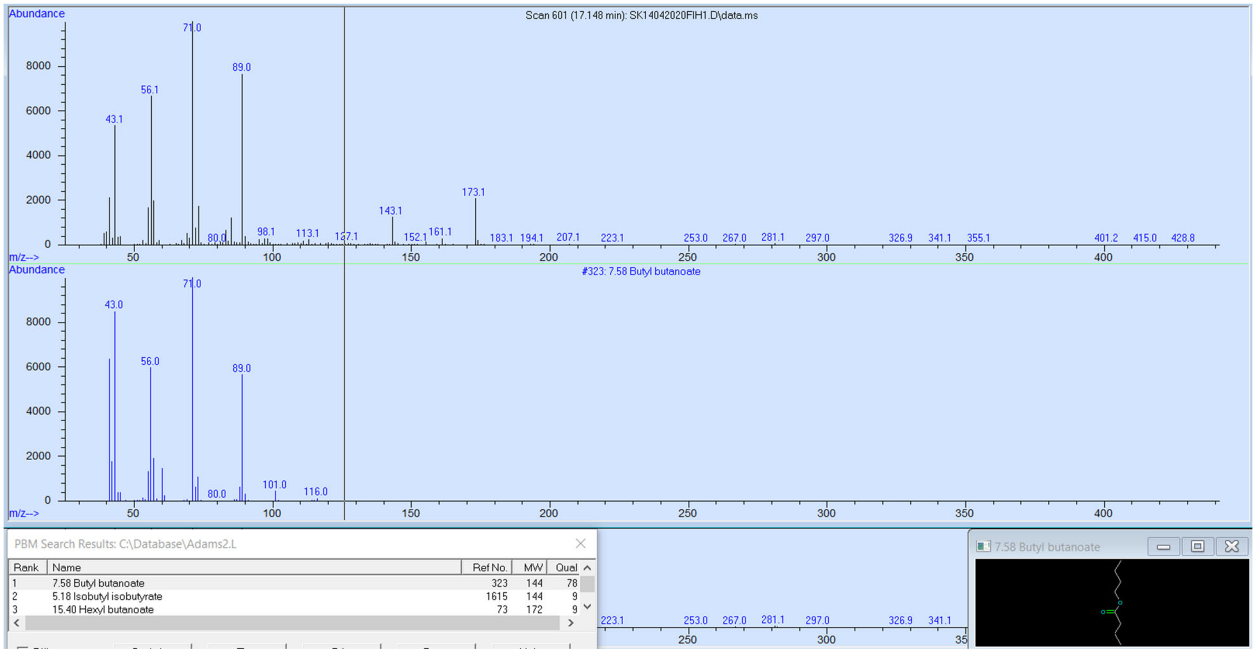


**Figure S24.** Mass spectrum of Propyl butanoate (**24**)

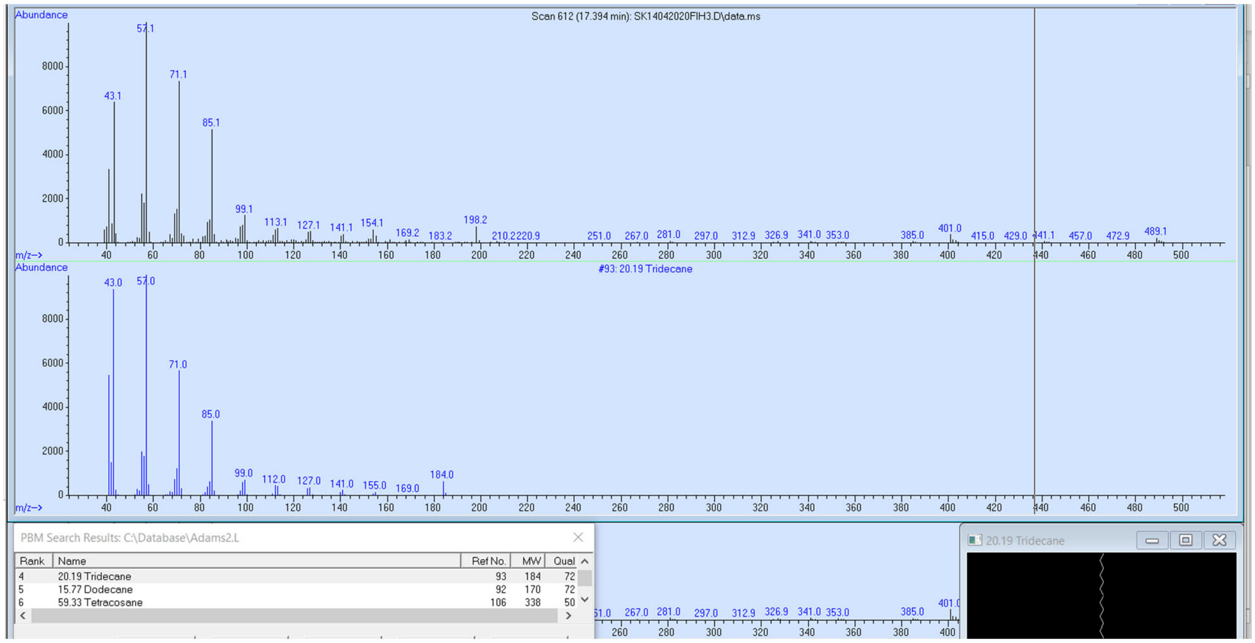


**Figure S25.** Mass spectrum of Tridecane (**25**)

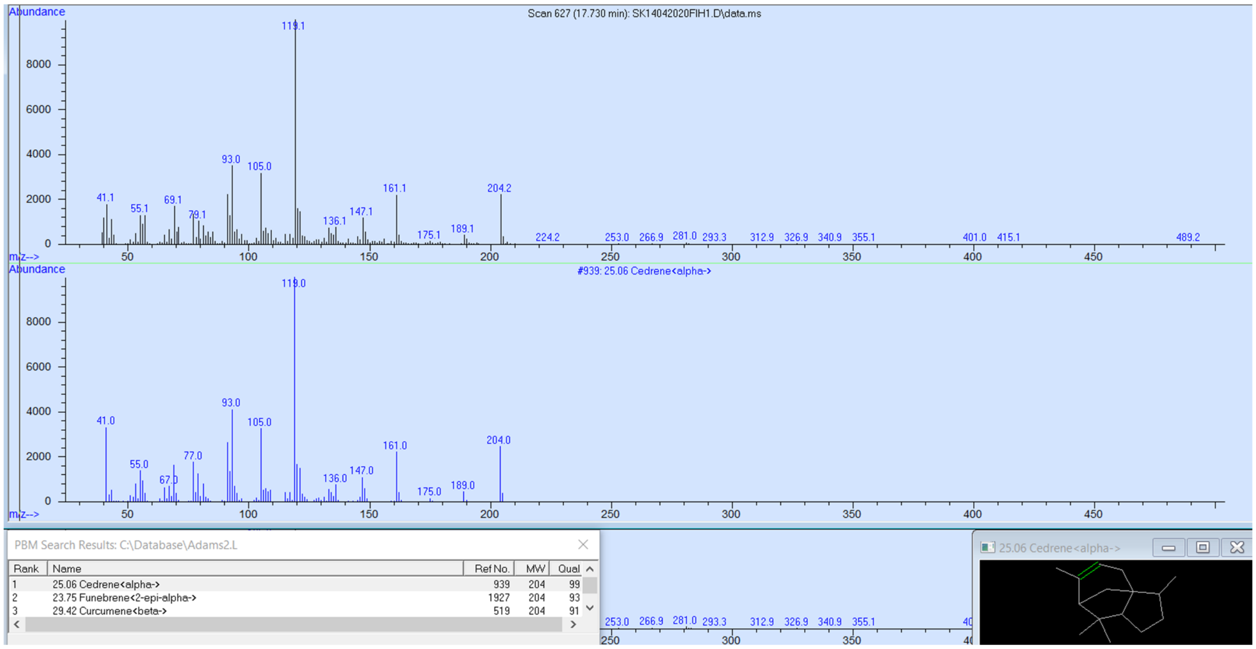


**Figure S26.** Mass spectrum of α-Cedrene (**26**)

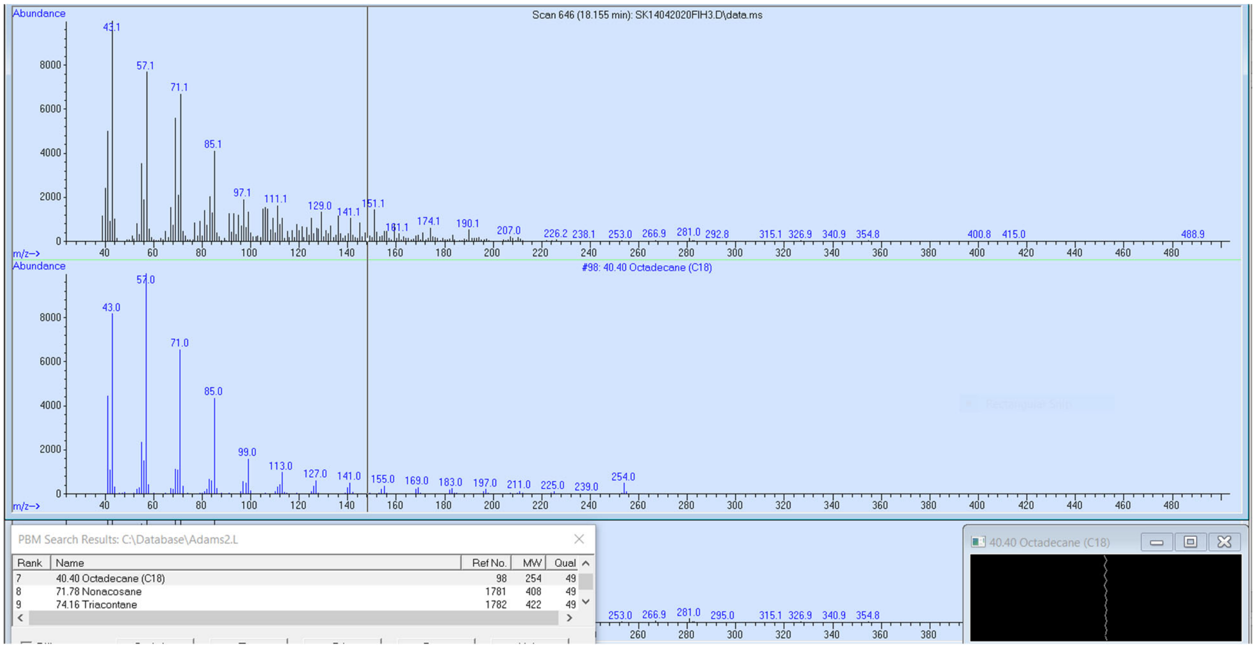


**Figure S27.** Mass spectrum of Octadecane (**27**)

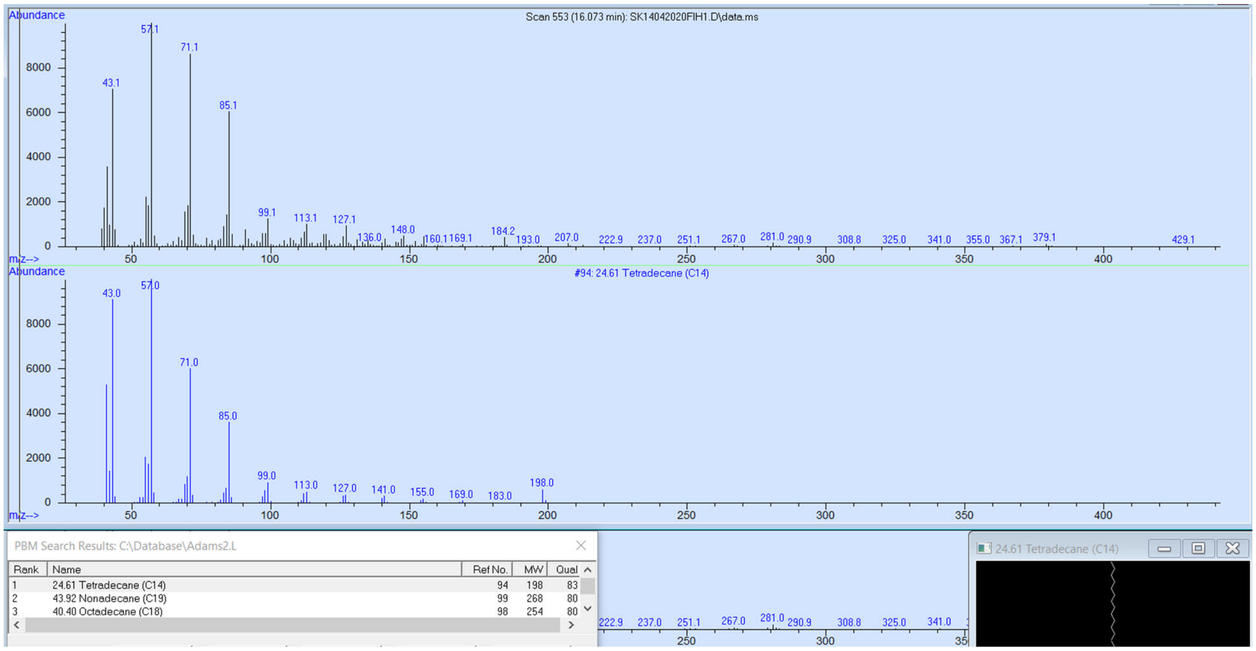


**Figure S28**. Mass spectrum of Tetradecane (**28**)

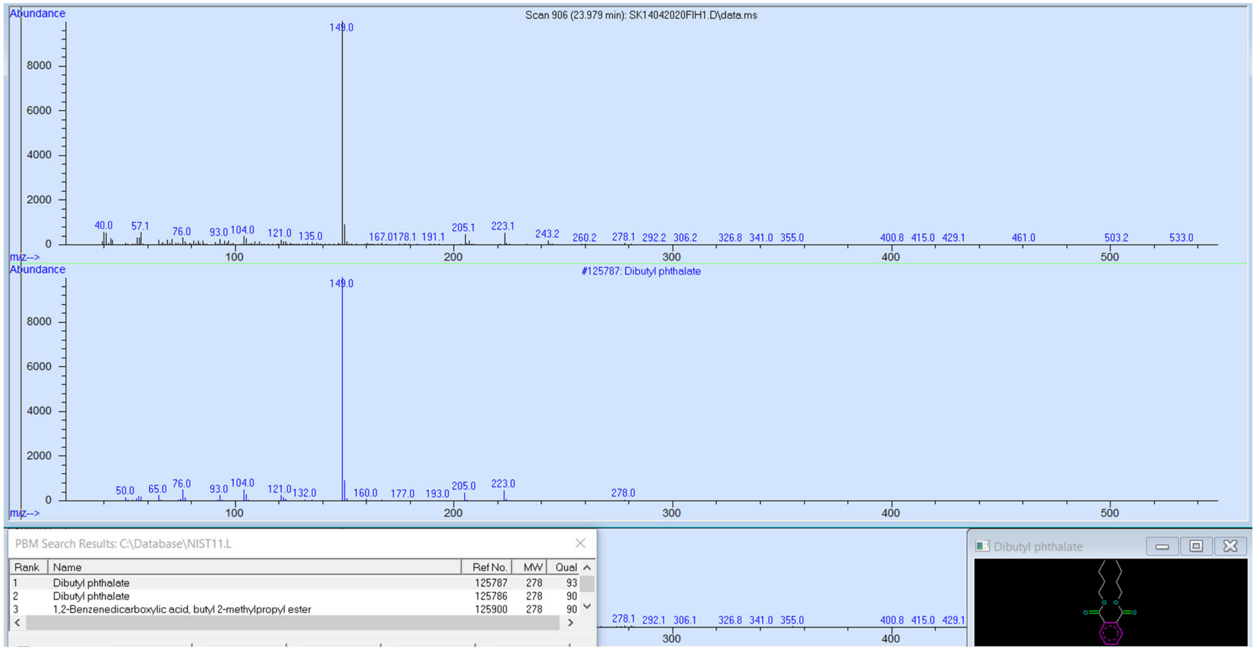


**Figure S29.** Mass spectrum of Dibutyl phthalate (**29**)

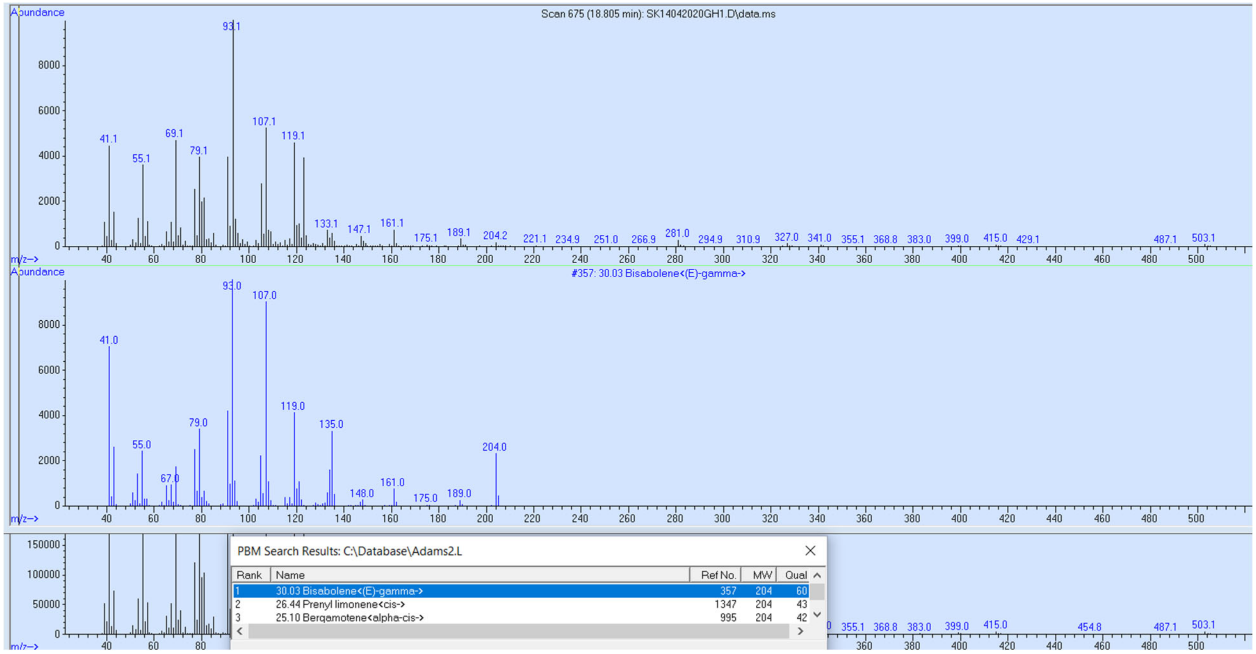


**Figure S30.** Mass spectrum of (*E*)-γ-Bisabolene (**30**)

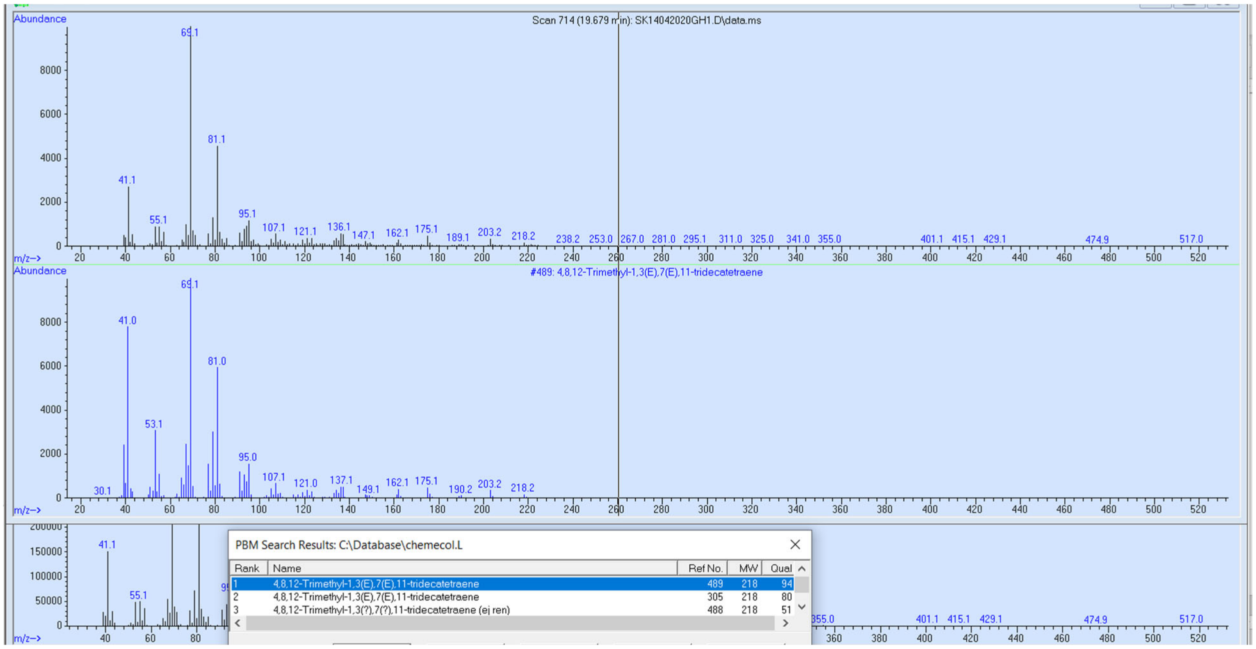


**Figure S31.** Mass spectrum of 4,8,12-Trimethyl-1,3*E*,7*E*,11-tridecatetraene (**31**)

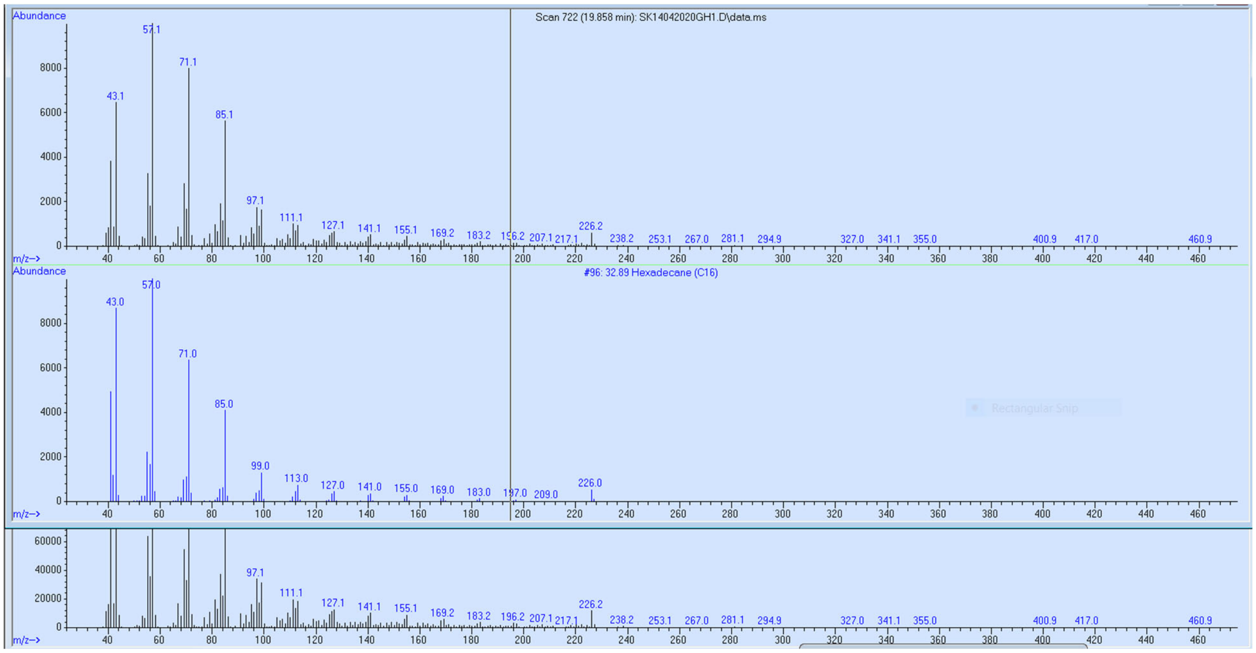


**Figure S32.** Mass spectrum of Sulfurous acid, pentylundecyl ester (**32**)

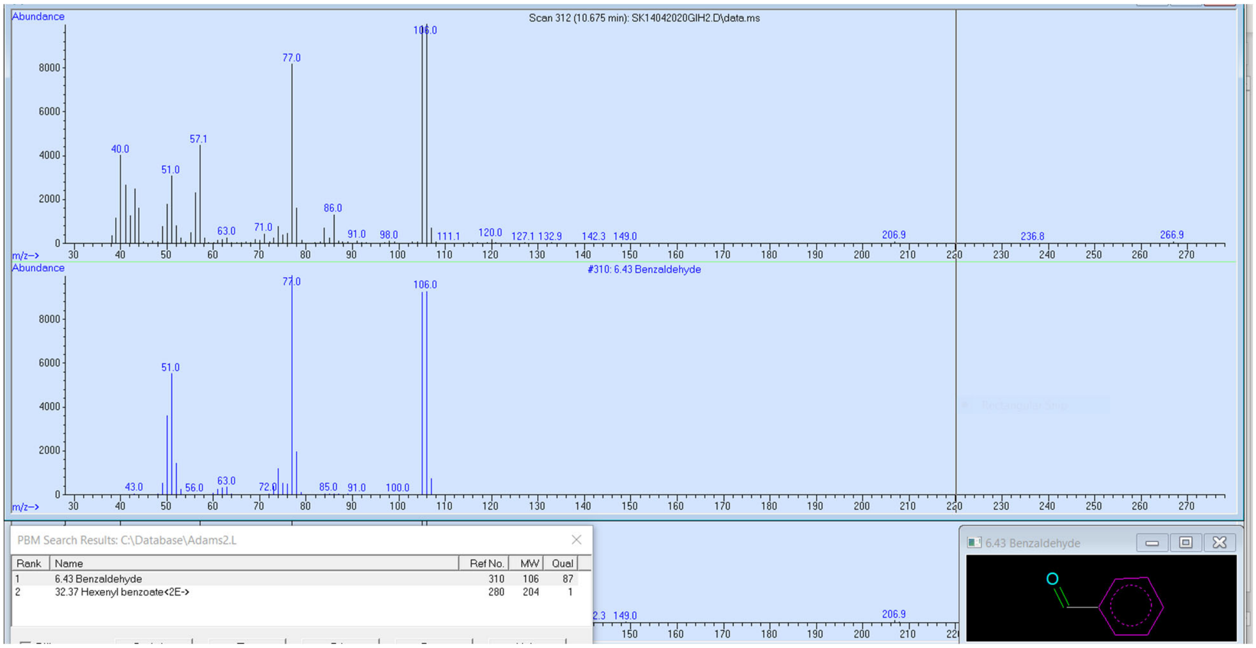


**Figure S33**. Mass spectrum of Benzaldehyde (**33**)

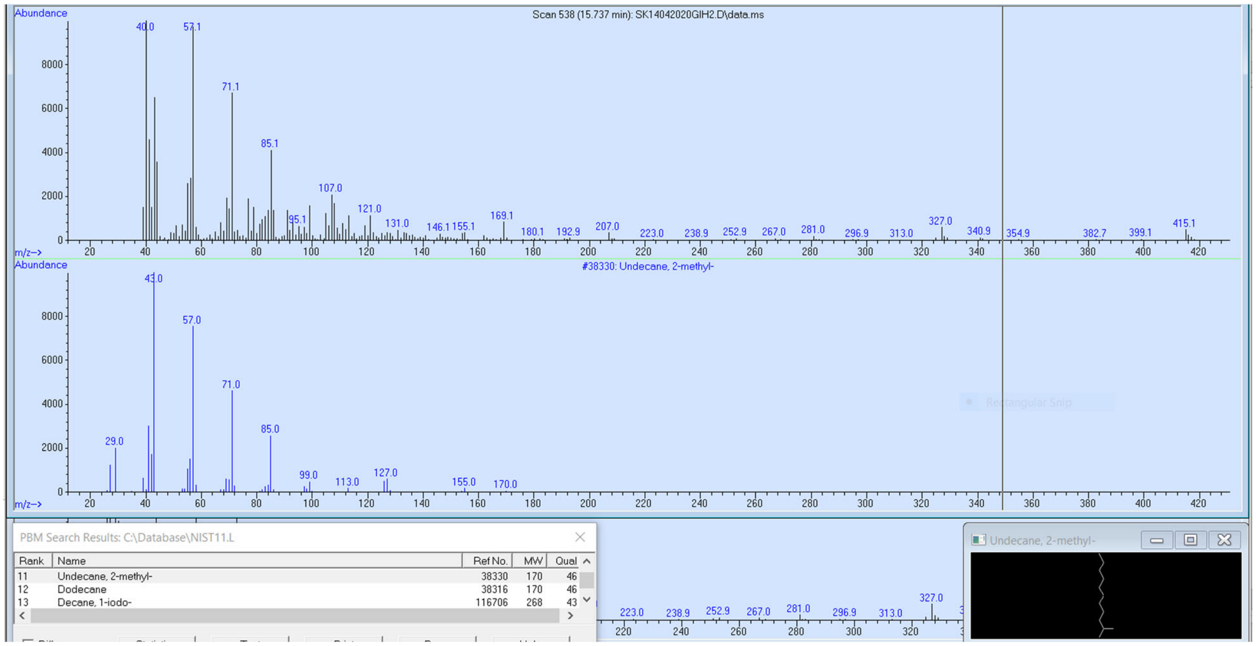


**Figure S34.** Mass spectrum of 5,7-Dimethylundecane (**34**)

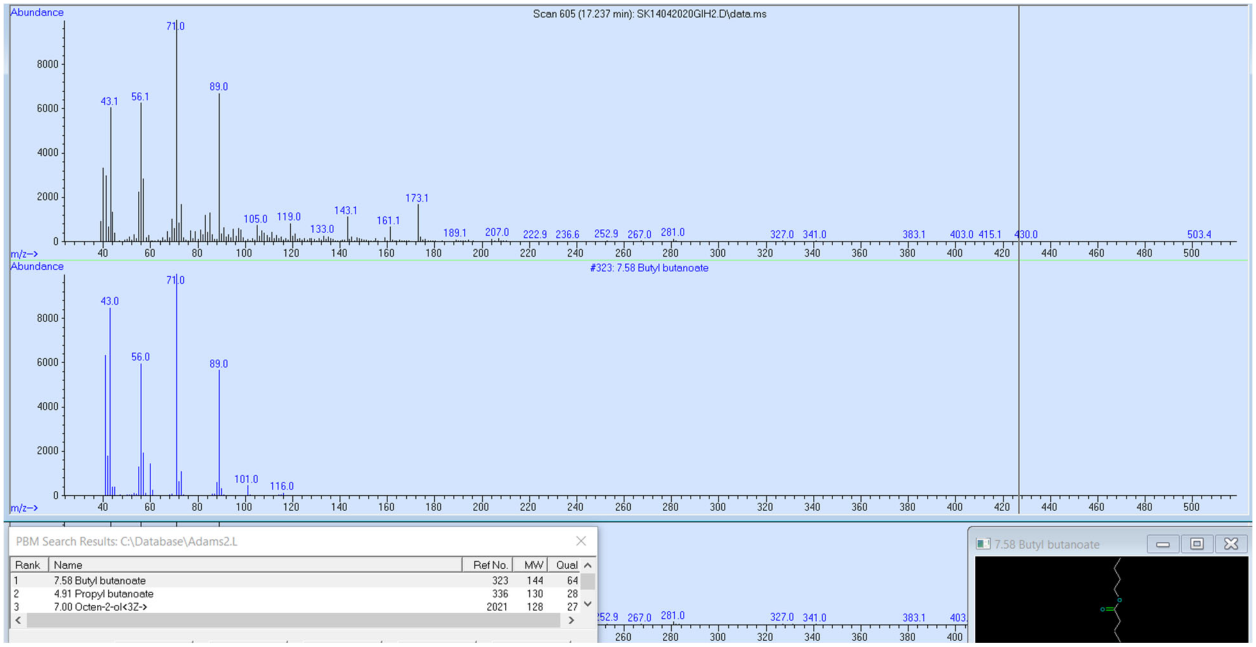


**Figure S35.** Mass spectrum of 2-Methyl-2-ethyl-3-hydroxyhexylpropanoate (**35**).
